# Increasing the Hindgut Carbohydrate/Protein Ratio by Cecal Infusion of Corn Starch or Casein Hydrolysate Drives Gut Microbiota-Related Bile Acid Metabolism To Stimulate Colonic Barrier Function

**DOI:** 10.1128/mSystems.00176-20

**Published:** 2020-06-02

**Authors:** Yu Pi, Chunlong Mu, Kan Gao, Zhuang Liu, Yu Peng, Weiyun Zhu

**Affiliations:** a Laboratory of Gastrointestinal Microbiology, Jiangsu Key Laboratory of Gastrointestinal Nutrition and Animal Health, College of Animal Science and Technology, Nanjing Agricultural University, Nanjing, China; b National Center for International Research on Animal Gut Nutrition, Nanjing Agricultural University, Nanjing, China; Dalhousie University

**Keywords:** nutrient availability, large intestine, gut microbiota, bile acid, gut health, piglets

## Abstract

Dietary high protein and low carbohydrate levels compromise colonic microbiota and bile acid metabolism, which underlies a detrimental gut environment. However, it remains unclear if the diet-induced changes in colonic health are due to a change in hindgut nutrient availability and what key intermediates link the microbe-epithelium dialogue. To specifically alter the hindgut nutrient substrate availability, here we used a cecally cannulated pig model to infuse corn starch and casein hydrolysate directly into the cecum to generate a stepwise change of carbohydrate/nitrogenous compound (C/N) ratio. Pigs were cecally infused daily with either saline (Control), corn starch (Starch), or casein hydrolysate (Casein) (*n* = 8 per group), respectively, for 19 days. After infusion, C/N ratios in colonic digesta were 16.33, 12.56, and 8.54 for the starch, control, and casein groups, respectively (*P < *0.05). Relative to the control group, casein infusion showed greater abundance of the bacteria (*Eubacterium*) capable of bile acid 7α-dehydroxylation (*baiJ*), higher levels of expression of bacterial genes encoding the *baiJ* enzyme, and higher levels of secondary bile acid (deoxycholic acid [DCA] and lithocholic acid [LCA]), while the starch infusion showed the opposite effect. Correspondingly, casein infusion downregulated expression of genes encoding tight junction proteins (ZO-1 and OCLD) and upregulated expression of genes encoding epidermal growth factor receptor (EGFR). The ratio of C/N was linearly related with the concentrations of DCA and LCA and gene expression levels of ZO-1, occludin, and EGFR. Caco-2 cell experiments further showed that DCA and LCA downregulated expression of genes involved in barrier function (ZO-1 and OCLD) and upregulated the gene expression of EGFR and Src. Inhibition of EGFR and Src could abolish DCA- and LCA-induced downregulation of ZO-1, indicating that DCA and LCA impair gut barrier function via enhancing the EGFR-Src pathway. These results suggest that the ratio of C/N in the large intestine is an important determinant of microbial metabolism and gut barrier function in the colon. The findings provide evidence that microbe-related secondary bile acid metabolism may mediate the interplay between microbes and gut barrier function.

**IMPORTANCE** High-fiber or high-protein diets could alter gut microbiota and health in the large intestine, but factors involved in the effects remain unclear. The present study for the first time demonstrates that the starch- and casein-induced C/N ratio in the hindgut is an important factor. Using the cannulated pig model, we found that the distinct C/N ratio induced by cecal infusion of corn starch or casein hydrolysate was linearly correlated with microbial metabolites (secondary bile acids) and tight junction proteins (ZO-1 and OCLD). Cell culture study further demonstrates that the gut microbial metabolites (DCA and LCA) could impair the intestinal barrier function via the EGFR-Src pathway. These suggest that DCA and LCA were key metabolites mediating microbe-epithelium dialogue when the hindgut C/N ratios were altered by cecal infusion of corn starch or casein hydrolysate. These findings provide new insight into the impact of C/N ratio in the large intestine on colonic health and provide a new framework for therapeutic strategy in gut health through targeted manipulation of hindgut microbiota by increasing the carbohydrate level in the large intestine.

## INTRODUCTION

Gut microbiota, especially in the large intestine, play an important role in gastrointestinal health and animal performance. Microbes need nutrients for their cell growth and activity, and proteins and carbohydrates are the two major nutrients; thus, diet composition can influence gut microbiota. For example, a high-protein diet increases the population of Clostridium leptum group in the colon of piglets (1); increases abundance of *Escherichia/Shigella* and *Streptococcus* and decreases that of *Ruminococcus*, *Akkermansia*, and Faecalibacterium prausnitzii in the colon of rats (2); and decreases the population of *Roseburia/*Eubacterium rectale group in human feces (3). Dietary high resistant starch or fiber decreases the relative abundance of *Clostridium* and *Bacteroidales* S24-7 group and increases the relative abundance of *Blautia*, *Ruminococcus*, and *Coprococcus* (4) or *Clostridium* cluster XIVa in colonic digesta and mucosa in pigs (5) and increases relative abundance of Ruminococcus bromii in human feces (6). Diet composition can affect the metabolic activities of the microbiota that adapts to the intestinal luminal environment (7). For example, resistant starch consumption could decrease fecal deoxycholic acid (DCA) concentration by 50% in human (8). In addition, compared with high-fiber diets, a high-protein diet could increase the levels of fecal DCA and lithocholic acid (LCA) in humans (9). Moreover, previous studies showed that a dietary protein source with different amino acid compositions could modulate plasma bile acid (BA) levels in rats (10, 11). In the host, about 5% of whole BAs escape the enterohepatic circulation and serve as the substrate for gut microbiota. Gut microbes can transform cholic acid (CA) and chenodeoxycholic acid (CDCA) via dehydrogenation by the BA 7α-dehydroxylase (*baiJ*) to DCA and LCA in the large intestine. Some strains of *Lactobacillus*, *Lachnospiraceae*, *Ruminococcaceae*, *Clostridiaceae*, *Eubacterium*, and *Peptostreptococcus* are 7α-dehydroxylase-active intestinal bacteria (carrying the *baiJ* gene) (12–14), pointing out that diet can affect gut BA metabolism by influencing the gut microbiota.

Dietary high fiber and high protein can differently regulate gut health. For example, resistant starch consumption could decrease colonic mucosal proliferation from 6.7% to 5.4% in humans (8). In addition, a fiber and resistant-starch diet could also enhance colonic barrier function by increasing mucin content and upregulating the expression of genes encoding mucin and claudins (15–17). In contrast, a high-protein diet has been linked with increased disease risk in the colon as reflected by elevated levels of carcinogens, such as ammonia, nitrosamines, biogenic amines, and BAs (9). BAs have emerged as important and pleiotropic signaling metabolites involved in the regulation of metabolism and inflammation through interacting with both microbiota and host receptors (18). However, how BA mediates the diet-induced changes of epithelial barrier function is still unclear.

The influence of diet on gut microbiota and epithelial responses may be due to the increased flow of undigested carbohydrates (C) or nitrogenous compounds (N) into the large intestine, resulting in different nutritional availability for the gut microbiota and intestinal environment (19). This seems reasonable, but the effects may be interfered by foregut metabolism. However, direct evidence is limited on whether the different responses of gut microbiota in the large intestine were caused by the C and N availability. Thus, a cannulated piglet model, which allows easy manipulation of the C and N availability at specific intestinal segments (20), is necessary to investigate the responses of gut microbiota and epithelium to directly increase C and N availability in the large intestine.

Therefore, we hypothesize that changing the ratio of C/N in the large intestine could modulate the colonic microbial community and microbial BA metabolism, which will affect colonic barrier function. To test this hypothesis, a cecal cannulated pig model was employed, considering that the pig has similar digestive physiology as humans (21) but allows easy handling and manipulation of C/N ratio at specific gut segments. To specifically change the ratio of C/N in the large intestine, corn starch or casein hydrolysate was infused into the cecum of the pigs. Furthermore, *in vitro* Caco-2 cell cultures were performed to explore the mechanistic role of microbe-related BAs in the gut barrier function. The colonic mucosal microbial composition, BA metabolism, and barrier function were determined, aiming to understand the impact of C/N ratio on the colonic barrier function and the possible mechanism of action by microbial metabolites.

## RESULTS

### Carbohydrate and nitrogenous compound levels in the colon and growth performance.

**FIG 1:**
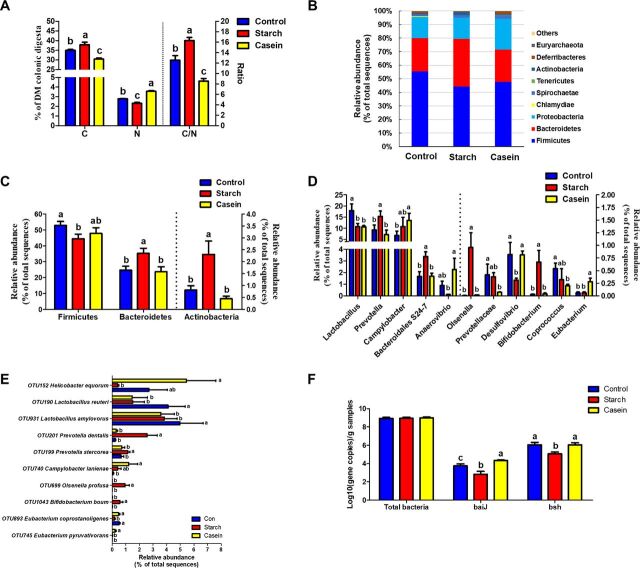
Main nutrient substrates in colonic digesta and colonic mucosa bacterial composition profiles (*n* = 8). (A) Contents of carbohydrates and nitrogenous compounds of colonic digesta (% of DM of digesta). (B) The relative abundance of phyla in the composition of colonic mucosa-associated bacteria of pigs. (C) Significantly changed bacteria at phylum level. (D) The representative significantly changed bacteria at the genus level in colonic mucosa of pigs. See Table S5 for the complete data set. (E) The representative phylotypes in colonic mucosa of pigs. See Table S6 for the complete data set. (F) The copy numbers of total bacteria and bacterial groups known to include genes encoding bile acid 7α-dehydroxylation (*baiJ*) and bile acid hydrolase (*bsh*). Data are presented as mean ± SEM. In-column values without a common letter significantly differ, *P* < 0.05 or *q *< 0.05. No letters above the columns means that there are no statistically significant differences, *P* > 0.05 or *q *> 0.05. Control, control group, pigs cecally infused with saline; Starch, starch group, pigs cecally infused with corn starch; Casein, casein group, pigs cecally infused with casein hydrolysate; C/N, the ratio of total carbohydrate and nitrogenous compounds; OTU, operational taxonomic unit.

After cecal infusion of corn starch (50 g/day) and casein hydrolysate (50 g/day), the ratio of carbohydrates and nitrogenous compounds (C/N) was 12.56, 16.33, and 8.65 for the control, starch, and casein groups, respectively (Fig. 1A). All pigs remained in good health with no clinical signs of diarrhea or health impairment throughout the experiment. Compared with the control group, the starch group showed a higher average daily feed intake and higher body weight gain, with a lower ratio of feed intake/weight gain throughout the study period. The casein group did not show any difference in the growth performance from the control (see Table S3 in the supplemental material). The weight of colonic tissue, the weight/length ratio, and relative weight were significantly greater in the starch group than in the control group and casein group (Table S4). There were no differences in pH value, colonic length, digesta weight, and digesta moisture content among the three groups (*P > *0.05).

### Colonic mucosal microbial composition.

High-throughput sequencing showed that the rarefaction curves plotting the number of sequences by the number of operational taxonomic units (OTUs) tended to approach the saturation plateau (Fig. S1A). Principal-coordinate analysis (PCoA) plots based on unweighted UniFrac distance showed a separation among the three groups with PCo1 and PCo2 at 22.88% and 14.10% of the explained variance, respectively (Fig. S1B). Unweighted UniFrac analysis revealed a trend of difference among the three groups (UniFrac score, 0.930; *P* = 0.088). The statistical estimates of species richness for 5,000-sequence subsets from each sample showed no differences in diversity indices (Shannon and inverse Simpson) and richness estimators (abundance-based coverage estimator [ACE] and Chao1) of the colonic mucosa bacteria among the three groups (Fig. S1C).

10.1128/mSystems.00176-20.1 FIG S1 Illumina MiSeq sequencing analysis of colonic mucosa bacteria of growing pigs among three treatment groups (*n* = 8). (A) Rarefaction curves. (B) Principal-coordinate analysis plots based on an unweighted UniFrac distances. (C) Diversity indices. (D) Effects of corn starch and casein hydrolysate infusions on average relative abundance of genus level (% of total sequences) in colonic mucosa. Data are presented as mean ± SEM. Control, control group, pigs cecally infused with saline; Starch, starch group, pigs cecally infused with corn starch; Casein, casein group, pigs cecally infused with casein hydrolysates; OTU, operational taxonomic unit; PC1, principal coordinate 1; PC2, principal coordinate 2; ACE, abundance-based coverage estimator. Download FIG S1, TIF file, 2.8 MB. Copyright © 2020 Pi et al. 2020 Pi et al. This content is distributed under the terms of the Creative Commons Attribution 4.0 International license.

*Firmicutes*, *Bacteroidetes*, and *Proteobacteria* were the three most dominating phyla (Fig. 1B). The starch group showed a lower relative abundance of *Firmicutes* than the control (44.38% versus 55.43%) and a greater relative abundance of *Bacteroidetes* (35.20% versus 24.67% and 23.7%) and *Actinobacteria* (2.30% versus 0.81% and 0.45%) compared with the control group and casein group, respectively (*q* < 0.05). However, the casein group showed no significant difference from the control group (Fig. 1C).

At the genus level, *Lactobacillus* (with abundance at 17.93%, 10.71%, and 10.59% in control, starch, and casein groups, respectively), *Prevotella* (9.48%, 17.86%, and 7.20% in control, starch, and casein groups, respectively), and *Campylobacter* (6.78%, 10.74%, and 13.55% in control, starch, and casein groups, respectively) were the dominant genera (Fig. S1D). Within the top 100 genera, 16 genera significantly responded to the starch infusion and 10 genera significantly responded to casein infusion, in comparison with the control (Fig. S2). Compared with the control group, the starch group had greater abundances of *Prevotella*, *Bacteroidales* S24-7 group, *Olsenella*, and *Bifidobacterium* and lower abundances of *Lactobacillus* and *Desulfovibrio* (*q* < 0.05) (Fig. 1D and Table S5). The casein group, in comparison with the control group, showed greater abundances of *Campylobacter* and *Eubacterium* and lower abundances of *Lactobacillus*, *Prevotellaceae*, and *Coprococcus* (*q* < 0.05).

10.1128/mSystems.00176-20.2 FIG S2 Heatmap of the dominant genus in colonic mucosa. The mean relative abundance was calculated using all the samples in control and treatment groups. For *q* value, red represents a significant increase between the two groups (*q *< 0.05), blue represents a significant decrease between the two groups (*q *< 0.05), and white represents no significant difference between the two groups (*q *> 0.05). See Table S4 for the complete data set. Con, control group, pigs cecally infused with saline; Starch, pigs cecally infused with corn starch; Casein, pigs cecally infused with casein hydrolysates. Abbreviations: Starch vs. Con, Starch group compared with Con group; Starch vs Casein, Starch group compared with Casein group; Casein vs Con, Casein group compared with Con group. Download FIG S2, TIF file, 2.8 MB. Copyright © 2020 Pi et al. 2020 Pi et al. This content is distributed under the terms of the Creative Commons Attribution 4.0 International license.

At the OTU level, compared with control group, the starch group mainly increased OTU201 Prevotella dentalis (99%), OTU199 Prevotella stercorea (98%), OTU699 Olsenella profuse (96%), OTU1043 Bifidobacterium boum (100%), and OTU10 Succinivibrio dextrinosolvens (98%) and decreased OTU190 Lactobacillus reuteri (100%), OTU931 Lactobacillus amylovorus (100%), and OTU893 Eubacterium coprostanoligenes (100%) (*q *< 0.05), while the casein group did not show significant difference in these bacteria (Fig. 1E, Fig. S3, and Table S6). The casein group, in comparison with the control group, mainly increased OTU740 Campylobacter lanienae (99%) and OTU745 Eubacterium pyruvativorans (100%) and decreased OTU190 Lactobacillus reuteri (99%) and OTU931 Lactobacillus amylovorus (100%) (*q *< 0.05), while the starch group did not affect these bacteria.

10.1128/mSystems.00176-20.3 FIG S3 Heatmap of the dominant OTUs in colonic mucosa. The mean relative abundance was calculated using all the samples in three treatment groups. See Tables S5 for the complete data set. Con, control group, pigs cecally infused with saline; Starch, starch group, pigs cecally infused with corn starch; Casein, casein group, pigs cecally infused with casein hydrolysates. Abbreviations: Starch vs. Con, Starch group compared with Con group; Starch vs Casein, Starch group compared with Casein group; Casein vs Con, Casein group compared with Con group. Asterisks indicate significant differences between the two groups. *, *q *< 0.05. Download FIG S3, TIF file, 2.8 MB. Copyright © 2020 Pi et al. 2020 Pi et al. This content is distributed under the terms of the Creative Commons Attribution 4.0 International license.

As MiSeq sequencing analysis can reflect only the relative abundance of bacteria, quantitative real-time PCR was performed to determine the completed 16S rRNA gene copies of bacteria in the colonic mucosa of pigs (Fig. 1F). Infusion of corn starch or casein hydrolysates had no effects on total bacteria (*P > *0.05). Compared with the control group, starch infusion significantly decreased the copy numbers of BA 7α-dehydroxylase (*baiJ*) and BA hydrolase (*bsh*) genes, while casein infusion significantly increased the copy number of the *baiJ* gene involved in conversion of primary to secondary BAs.

### Microbial metabolites in colonic digesta.

**FIG 2:**
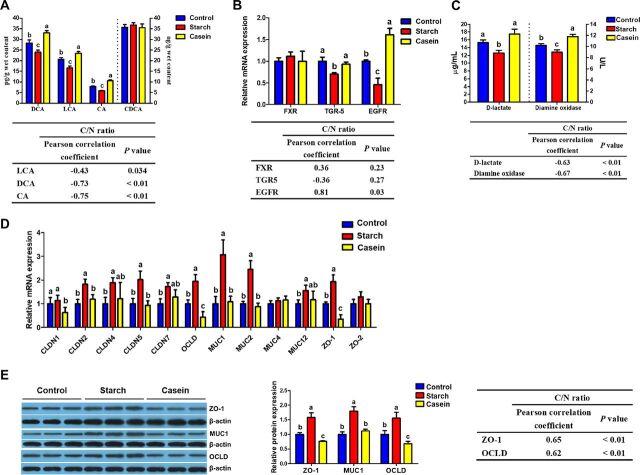
Responses of microbial metabolite profiles and colonic mucosa barrier function of pigs toward treatments (*n* = 8). (A) Bile acid profiles and their correlations with the ratio of total carbohydrate and nitrogenous compounds (C/N ratio). (B) mRNA expression of genes related to bile acid receptors and their correlations with the C/N ratio. (C) Responses of serum concentration of d-lactate and activity of diamine oxidase and their correlations with the C/N ratio. (D) mRNA expression of genes related to mucin and tight junction proteins. (E) Relative protein levels of ZO-1, MUC1, and OCLD and their correlations with the C/N ratio. Data are presented as means ± SEM. In-column values without a common letter significantly differ, *P* < 0.05. No letters above the columns means that there are no statistically significant differences, *P* > 0.05. Control, control group, pigs cecally infused with saline; Starch, starch group, pigs cecally infused with corn starch; Casein, casein group, pigs cecally infused with casein hydrolysate. CA, cholic acid; CDCA, chenodeoxycholic acid; DCA, deoxycholic acid; LCA, lithocholic acid; CLDN, claudin; OCLD, occludin; MUC, mucin; ZO, zonula occludens; FXR, farnesoid X receptor; TGR5, G-protein-coupled receptor; EGFR, epidermal growth factor receptor.

Gut microbiota can participate in BA metabolism by transforming the primary BAs (e.g., CA and CDCA) to secondary BAs (e.g., DCA and LCA). As for BA profile (Fig. 2A), compared with control group, the starch group had higher concentrations of DCA, LCA, and CA, whereas the casein group had lower concentrations. Moreover, the concentrations of DCA, LCA, and CA were negatively correlated with the ratio of C/N. Hence, these results indicate that starch infusion and casein infusion led to opposite shifts in BA profile in the colon.

### Colonic mucosal barrier function and goblet cell characteristics.

Farnesoid X receptor (FXR), G-protein-coupled receptor (TGR5), and epidermal growth factor receptor (EGFR) are important receptors of BAs, which can regulate gut barrier function. Compared with the control group, the starch group downregulated gene expression of *TGR5* and *EGFR* (*P < *0.05), while the casein group upregulated gene expression of *EGFR* (*P < *0.05) (Fig. 2B). The gene expression of *EGFR* was positively correlated with the ratio of C/N. The serum d-lactate and diamine oxidase (DAO) serve as two well-established markers of intestinal integrity. Compared to the control group, the starch group markedly decreased the concentration of d-lactate and the activity of DAO, whereas the casein group markedly increased the activity of DAO (*P < *0.05) (Fig. 2C). Linearly negative relationships were found between the concentration of d-lactate and the activity of DAO with the ratio of C/N. Gut microbiota and metabolites can regulate epithelial gene expression and cell populations. Therefore, we analyzed the expression of genes involved in mucosal barrier function and the numbers of goblet cells. Mucins (MUC) reflect the chemical barrier function, and claudin (CLDN), occludin (OCLD), and zonula occludens-1 (ZO-1) reflect the mechanical barrier function. Compared with the control group, the starch group upregulated gene expression for *MUC1*, *MUC2*, *MUC12*, *CLDN 2*, *CLDN4*, *CLDN5*, *CLDN7*, *OCLD*, and *ZO-1* (*P* < 0.05) but showed no effect on *CLDN1*, while the casein group downregulated gene expressions for *ZO-1*, *OCLD*, and *CLDN1* (*P* < 0.05) (Fig. 2D). These results of gene expressions were further validated by analysis of protein expression through Western blotting (Fig. 2E). It is also interesting that the gene expression of ZO-1 and OCLD was positively correlated with the ratio of C/N in colon.

**FIG 3:**
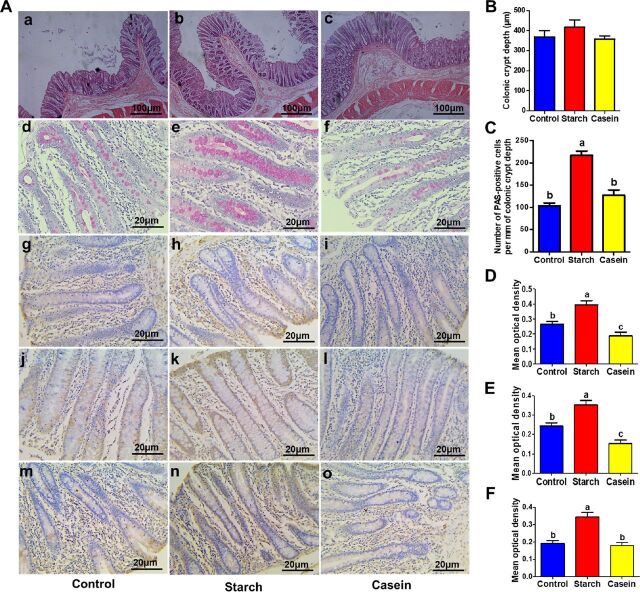
Responses of colonic morphology; mucus-producing cells; and OCLD, ZO-1, and MUC1 protein levels in the colon of pigs to control, starch, and casein treatments (*n* = 8). (A) Histological examination of colonic sections stained with hematoxylin-eosin (a to c) and PAS (d to f) and representative images of OCLD (g to i), ZO-1 (j to l), and MUC1 (m to o) proteins in the colon. The OCLD, ZO-1, and MUC1 proteins in colon tissue were measured by immunohistochemical methods. OCLD-, ZO-1-, and MUC1-positive cells were stained in brown and present on the cell membrane. The bigger brown area and deeper color represent the higher target protein levels. (B) Colonic crypt length. (C) Number of PAS-positive cells per length of colonic crypt. (D to F) Mean optical density of OCLD (D), ZO-1 (E), and MUC1 (F) proteins in the colon. Data are presented as mean ± SEM. In-column values without a common letter significantly differ, *P* < 0.05. Control, control group, pigs cecally infused with saline; Starch, starch group, pigs cecally infused with corn starch; Casein, casein group, pigs cecally infused with casein hydrolysate. PAS, periodic acid-Schiff stain; ZO-1, zonula occludens 1; MUC, mucin; OCLD, occludin.

Starch infusion and casein infusion also showed different effects on the colonic morphology (Fig. 3A). Both the starch group and casein group had no effect on colonic crypt depth compared with the control group (Fig. 3B). However, compared with the control group, the starch group showed a greater number of goblet cells (*P < *0.05), while the casein group had no difference (Fig. 3C). The OCLD, ZO-1, and MUC1 protein expression in colonic tissue was also determined by immunohistochemical methods. The OCLD-, ZO-1-, and MUC1-positive cells were stained in brown and present on the cell membrane. The bigger brown area and deeper color represent the higher target protein levels (Fig. 3A). Compared with the control group, the starch group upregulated protein expression for OCLD (Fig. 3D) and ZO-1 (Fig. 3E) (*P* < 0.05), while the casein group downregulated gene expression for OCLD and ZO-1 (*P* < 0.05). The starch group upregulated protein expression for MUC1 (*P* < 0.05), while the casein group had no evident effect (Fig. 3F).

### Effects of secondary bile acids on the gut barrier function on Caco-2 cells.

**FIG 4:**
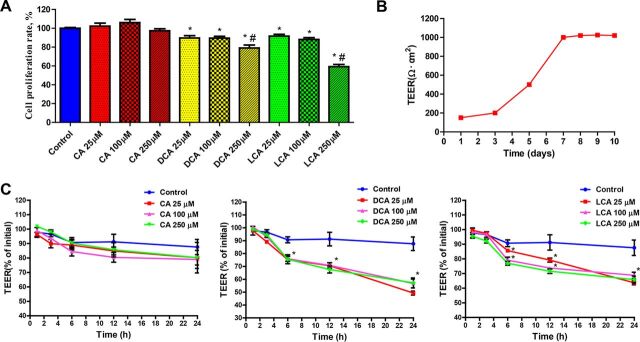
(A) Effects of DCA and LCA on cell proliferation of Caco-2 cells (*n* = 3). (B) The time to establish monolayer epithelial cells using Caco-2 cell lines. (C) Effects of DCA and LCA on transepithelial electrical resistance (TEER) of Caco-2 cells (*n* = 3). Data are presented as means ± SEM. *, compared with control, *P* < 0.05; #, compared with their two relative lower-dose groups, *P* < 0.05. CA, cholic acid; DCA, deoxycholic acid; LCA, lithocholic acid.

**FIG 5:**
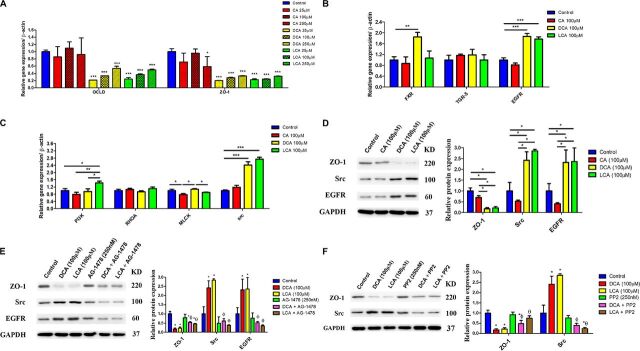
(A to C) Effects of selected bile acid on barrier function (A)-, bile acid receptor (B)-, and epithelial barrier function regulation (C)-related gene expression of Caco-2 cells (*n* = 3). (D) Effects of selected bile acid on the relative protein levels of ZO-1, Src, and EGFR (*n* = 3). GAPDH was used as an internal control. (E) Effects of selected bile acid and EGFR-specific inhibitor (AG-1478) on the relative protein levels of ZO-1, Src, and EGFR (*n* = 3). GAPDH was used as an internal control. (F) Effects of selected bile acid and Src family kinase inhibitor (PP-2) on the relative protein levels of ZO-1 and Src (*n* = 3). GAPDH was used as an internal control. Data are presented as mean ± SEM. *, compared with control, *P* < 0.05; **, compared with control, *P* < 0.01; ***, compared with control, *P* < 0.001; Ф, compared with DCA group, *P* < 0.05; θ, compared with LCA group, *P* < 0.05. CA, cholic acid; DCA, deoxycholic acid; LCA, lithocholic acid; OCLD, occludin; ZO-1, zonula occludens 1; FXR, farnesoid X receptor; TGR5, G-protein-coupled receptor; EGFR, epidermal growth factor receptor; GAPDH, glyceraldehyde-3-phosphate dehydrogenase; PI3K, phosphatidylinositol 3-kinase; RHOA, ras homolog gene family, member A; MLCK, myosin light chain kinase.

To determine whether DCA and LCA affect the integrity of intestinal epithelium, we examined the effect of DCA and LCA on epithelial permeability by measuring the transepithelial electrical resistance (TEER) in Caco-2 cells. Compared with the control group, treatments with DCA and LCA at three different doses inhibited the proliferation of epithelial cells (Fig. 4A), but CA had no effect (*P* > 0.05). After 7 days of cell culture, the stable TEER value remains stable (Fig. 4B), suggesting that cells can form monolayer epithelial cells after 7 days of culture. In addition, in comparison with the negative control, the TEER value was much smaller in Caco-2 cells treated with elevated levels of DCA and LCA but remained unchanged with CA treatment (*P* > 0.05) (Fig. 4C). Moreover, compared with the control group, DCA and LCA showed lower mRNA expression of genes encoding ZO-1 and OCLD (Fig. 5A), while CA showed no differences. In the gut, several receptors of BA, such as nuclear receptors farnesoid X receptor (FXR; NR1H4), G-protein-coupled receptor (TGR5) (22), and EGFR (23) may regulate gut barrier function. The gene expression results of these BA receptors are shown in Fig. 5B. Compared with the control group, DCA and LCA showed greater expression of the EGFR gene, while CA had no difference. These results suggest that DCA and LCA impair the gut barrier function via EGFR. Epithelial tight junction (TJ) is regulated by several intracellular signaling molecules and kinases, such as RHO kinase, MLCK, phosphatidylinositol 3-kinase (PI3K) signaling (24), and Src (25), which have been demonstrated to regulate TJ protein expression. Further analysis of these genes’ expression showed that treatments with DCA and LCA showed higher expression of the *src* gene, a potential target gene of EGFR (23), compared with the control group, while CA had no difference (Fig. 5C). In addition, the results of protein expression of EGFR, Src, and ZO-1 were consistent with mRNA expression of these genes (Fig. 5D). Using selective inhibitors, we showed that pretreatment with AG-1478, an EGFR-specific inhibitor, was able to abolish DCA- and LCA-induced upregulation of Src expression and downregulation of ZO-1 expression (Fig. 5E). In addition, we also found that pretreatment with PP-2, a Src family kinase inhibitor, was also able to abolish BA-induced downregulation of ZO-1 expression (Fig. 5F). These results indicated that secondary BAs (DCA and LCA) impair gut barrier function via the EGFR-Src pathway.

## DISCUSSION

High-fiber or high-protein diets can affect gut microbiota in the large intestine and colonic health (2, 4, 17). It is likely that these diets increased the concentrations of carbohydrates or nitrogenous compounds in the large intestine, consequently changing microbial composition and metabolism in the colon. The present study, by cecal infusion of corn starch or casein hydrolysate, for the first time investigated the responses of colonic mucosal microbiota and gut barrier function to the different hindgut C/N ratios. The infusion of corn starch or casein hydrolysate altered hindgut C/N ratios and differently shifted the microbial composition and its metabolites (secondary BAs) and led to opposite impacts on epithelial barrier function, with the C/N ratio linearly correlated with the concentrations of secondary BAs (DCA and LCA) and the expression of genes related to gut barrier function (mucin and tight junction). Cell culture study further validated that DCA and LCA could impair the gut barrier function via the EGFR-Src pathway. These findings suggest that the carbohydrate/nitrogenous compound ratio induced by cecal infusion of corn starch and casein hydrolysate in the hindgut is a crucial factor influencing the colonic health. The secondary BAs are important mediators in the interplay between gut microbiota and gut epithelial barrier function.

### Hindgut C/N ratio as a potential determinant of microbial composition and bile acid metabolism.

Dietary carbohydrate and protein levels can influence both the gut microbiota composition and the microbial fermentation process. In the present study, starch infusion (higher C/N ratio) mainly increased the abundances of *Prevotella*, *Bacteroides*, *Olsenella*, and *Bifidobacterium* group compared with the control group. *Prevotella* and *Bacteroides* have been reported to play a role in starch degradation (26). *Bifidobacterium* species are also known for their amylolytic activity in the gut of pigs (27). In contrast, casein infusion (lower C/N ratio) increased *Campylobacter* and *Eubacterium* and decreased *Ruminococcaceae* UCG-014, *Coprococcus*, and unclassified *Prevotellaceae*. *Campylobacter* and *Eubacterium* (Eubacterium pyruvativorans) have been reported as dominant protein fermenters, which could deaminate amino acids to produce ammonia, amines, or caproate (28, 29). The *Ruminococcaceae* family, *Coprococcus*, and *Prevotellaceae* are important fibrolytic or polysaccharide-degrading bacteria in the guts of mammals (30, 31). Thus, the decrease in the abundance of polysaccharide degradation bacteria in the casein group may be a direct consequence of the insufficient substrate to support growth.

A novel finding in the present study is that increasing the C/N ratio by starch infusion decreased secondary BA concentrations (mainly DCA and LCA) in the colon, whereas reducing the C/N ratio by casein infusion increased secondary BA concentrations compared with the control group. Previous studies reported that a high-protein diet intake could increase DCA and LCA levels in human feces compared with high-fiber diets (9, 32), and resistant-starch consumption could decrease fecal DCA concentration by 50% (8). Studies also demonstrated that the starch fermentation could inhibit secondary BAs generated by colonic microbes derived from humans (33). These reports suggest that a high level of carbohydrates in the large intestine after high-fiber diets or resistant-starch diets can result in lower levels of DCA or LCA. DCA and LCA are generated from primary BAs (CA and CDCA) by the microbial *baiJ* enzyme (34). Bacterial species within the genus *Eubacterium* have been well recognized to be able to produce the *baiJ* enzyme (35). In the present study, we found that increasing nitrogenous compound levels increased both the relative abundance of *Eubacterium* (Eubacterium pyruvativorans) and the gene expression of microbial *baiJ*, which could explain the higher levels of DCA and LCA in colon after casein hydrolysate infusion.

### Lower C/N ratio in hindgut could impair the colonic mucosal barrier function.

d-Lactate and DAO serve as two well-established markers of intestinal integrity, as they are normally present at very low levels in blood (36, 37). Tight junction (TJ) proteins (ZO-1 and OCLD) are main transmembrane and nonmembrane proteins that form intercellular junctions between the epithelial cells, thus regulating paracellular barrier properties in epithelia (38). Thus, the decrease in d-lactate and DAO levels parallels the upregulation of colonic mucosal protein expression of ZO-1 and OCLD in the starch group compared with the control group, suggesting that the colonic barrier function was enhanced after cecal corn starch infusion. In contrast, the increase of DAO level and the downregulation of OCLD and ZO-1 in the casein group reflect the changes in intestinal integrity, pointing out that the colonic barrier function was damaged after cecal casein hydrolysate infusion.

The damage to gut barrier function after casein hydrolysate infusion could result from both the effect of increased N level and the effect of decreased C level in the large intestine. On the one hand, a high level of nitrogenous compounds in the gut can promote protein fermentation and increase the concentrations of the harmful *N*-nitroso compounds, which can impair gut barrier function and adversely affect immune homeostasis (2, 3). On the other hand, a displacement of carbohydrate from the intestine can also impair the gut barrier function because of the lack of microbiota-accessible carbohydrate and carbohydrate fermentation; the consequent decrease of microbial fermentation products (short-chain fatty acids [SCFAs]) can compromise the energy source for intestinal epithelial cells (39, 40). In our present study, we found that cecal infusion of casein hydrolysate decreased the hindgut C/N ratio by increasing the level of N and also decreasing the level of C, parallel to the gut barrier function impaired. When N displaces C, the excessive N can enhance the protein fermentation and increase some detrimental microbial metabolites (for example, secondary bile acid), and the reduced carbohydrate can lead to decreased microbial fermentation products (SCFAs), which could further impair the gut barrier function. In addition, the results of this study further demonstrated that the ratio of hindgut C/N is linearly related to expression of genes involved in gut barrier function (ZO-1 and OCLD), which reinforces that the C/N ratio is an important determinant that influences the gut barrier function and health. Therefore, higher levels of carbohydrates in the large intestine (higher C/N ratio) could enhance the gut barrier function, whereas higher levels of nitrogenous compounds (lower C/N ratio) could impair the gut barrier function.

### Secondary bile acids (DCA and LCA) compromise gut barrier function via EGFR-Src signaling.

**FIG 6:**
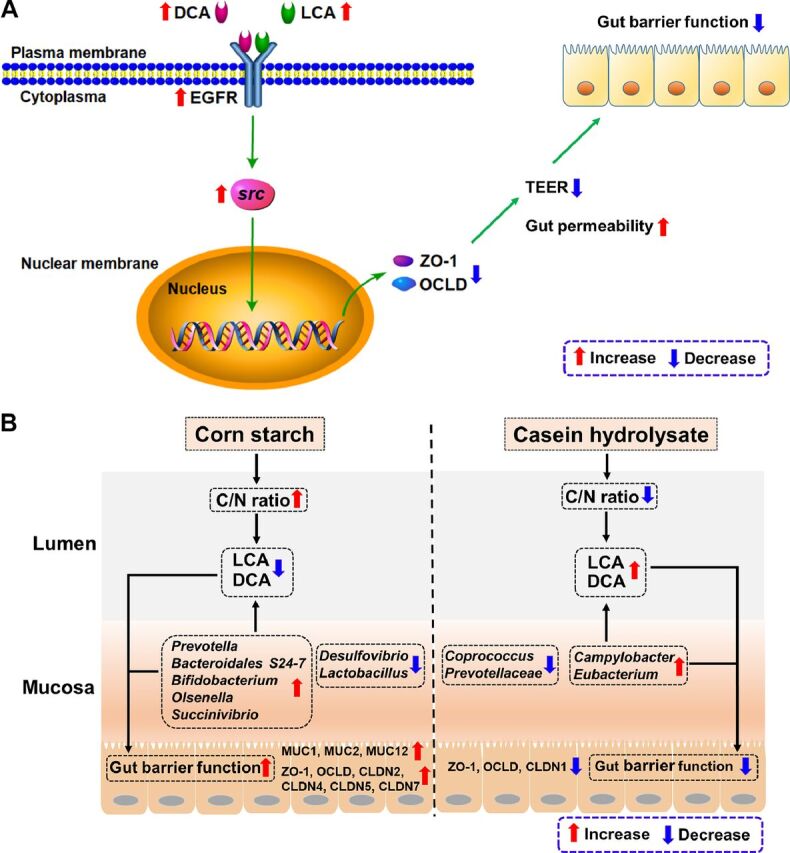
(A) The possible mechanisms of DCA and LCA on barrier function of Caco-2 cells. (B) Proposed model for the opposite effects of higher or lower ratios of C/N in the large intestine on colonic mucosal microbiota, microbial metabolites, and barrier function gene expression by cecal infusion of corn starch or casein hydrolysate, respectively. C/N, carbohydrates/nitrogenous compounds; DCA, deoxycholic acid; LCA, lithocholic acid; CLDN, claudin; OCLD, occludin; MUC, mucin; ZO-1, zonula occludens 1; EGFR, epidermal growth factor receptor; TEER, transepithelial electrical resistance.

In the present study, we found that the changes of the barrier function are accompanied by the changes of the secondary BAs (DCA and LCA), which are all linearly related to the C/N ratio, indicating that DCA and LCA may participate in the process of intestinal barrier damage under low hindgut C/N ratio. A previous study showed that the secondary BAs, DCA and LCA, could increase paracellular permeability in the Caco-2 cell model in a dose-dependent manner (50 to 250 μM) (41). In addition, Raimondi et al. showed that DCA (50 μM) could decrease TEER and increase paracellular permeability via EGFR activation in the Caco-2 cell model (23). In our present study, both DCA and LCA (25 to 250 μM) could downregulate the ZO-1 protein expression and decrease TEER value via EGFR-Src signaling activation in the Caco-2 cell line model (Fig. 6A). Thus, in our cannulated pig study, the downregulated gene expression of gut barrier function genes (*OCLD* and *ZO-1*) after casein hydrolysate infusion may be explained by the increased secondary BAs (DCA and LCA). Collectively, secondary BAs in the large intestine may serve as a key factor linking gut microbiota-colonic mucosal barrier function under different hindgut C/N ratios.

### The hindgut C/N ratio induced by cecal corn starch and casein hydrolysate infusion as an important factor influencing microbial BA metabolism and colonic health with secondary bile acid linking microbiota and gut barrier function: a proposed model.

Carbohydrates and proteins are the two major substrates for intestinal microbial metabolism. The current study demonstrated that increasing hindgut carbohydrate level by cecal corn starch infusion decreased the concentrations of DCA and LCA and promoted the expression of tight junction proteins (*OCLD* and *ZO-1*) in the porcine colon, while casein infusion showed opposite alterations. Cell culture study further demonstrated that DCA and LCA could impair the gut barrier function via the EGFR-Src pathway. The novel finding that the C/N ratio linearly correlated with secondary BAs (DCA and LCA) in colon and tight junction-associated gene expression (*OCLD* and *ZO-1*) suggests that the ratio of C/N is an important factor influencing microbial BA metabolism and colonic health. Comprehensively, we propose a model to depict the ratio of C/N in the colon influencing colonic barrier function with DCA and LCA linking the gut microbiota and gut barrier function (Fig. 6B). Specifically, a high level of carbohydrates (high C/N ratio) in the large intestine enhances colonic mucosal barrier function by decreasing the potential harmful microbial metabolites (secondary BAs, DCA and LCA), while high levels of nitrogenous compounds (low C/N ratio) are detrimental to colonic mucosal barrier function by increasing the concentrations of secondary BAs (DCA and LCA). The changes of microbial metabolites corresponded to the alterations in bacteria, such as the increased abundance of *Bacteroidales* S24-7 and *Bifidobacterium* with higher C/N ratio and the increased abundance of *Eubacterium* with lower C/N ratio.

This study demonstrates opposite impacts of increasing the carbohydrate or nitrogenous compound availability on the gut microbiota and health in the large intestine; this provides mechanistic insight into the colonic health due to the different C/N ratios that could be caused by high-fiber or high-protein diets. In addition, our findings showed that the gut microbiota, microbial BA metabolism, and gut barrier function showed parallel changes along with the C/N ratio alterations; this may provide potential clinical implications for improving gut health. Further, corresponding C/N ratios were also observed in feces in the present study (data not shown). Clinical research showed that the patients with colonic tumors had significantly higher levels of DCA and LCA in the aqueous extract of their feces than did healthy people (42). Therefore, both the ratio of C/N and the concentration of secondary BAs (DCA and LCA) in the feces, which are feasible to measure, may be a good indication of the health status in the large intestine. Our findings also imply that regulating the microbial BA metabolism in the large intestine by increasing carbohydrate level in the large intestine through dietary manipulation (e.g., resistant starch or fermentable fibers) is a potential approach for improving gut health.

## MATERIALS AND METHODS

### Ethical approval.

The experimental protocol and procedures for the care and treatment of the pigs were approved by the Animal Care and Use Committee of Nanjing Agricultural University (approval number SYXK 2018-0071).

### Animals and experimental procedures.

A total of 24 male 42-day-old cross-bred Duroc × (Landrace × Large White) weaned piglets, weighing 12.08 ± 0.28 kg, were individually housed in stainless steel metabolism cages (height, 0.85 m; length, 0.70 m; width, 0.70 m) with a nipple drinker and feeder and temperature controlled at 25°C. After a 7-day adaptation, pigs were fasted for 12 h prior to fitting a simple T-cannula in the cecum, approximately 5 cm cranial to the ileocecal sphincter. A comprehensive description of the surgical procedure and postoperative care has been given previously (20). Before surgery and during a 14-day recuperation period, the pigs were fed a 23% CP commercial starter diet. All pigs were fed individually, and water was provided *ad libitum*. After the 14-day recuperation, the pigs were allotted to experimental treatments according to a completely randomized design. The total duration of the experiment was 19 days, during which the pigs were infused 3 times per day (at 8:00, 12:00, and 20:00, respectively) with 10 ml sterile saline (control), 10 ml corn starch (Xinmao Group, Zhucheng, China) (50 g/day) suspended in sterile saline (starch group), or 10 ml casein hydrolysate (Qingdao Hope Bio-Technology Co. Ltd., Qingdao, China) (50 g/day) suspended in sterile saline (casein group). The ingredients and chemical composition of the basal diets are presented in Table S1 in the supplemental material. The diets were formulated to meet or exceed the nutrient demand according to the National Research Council (NRC) (50) standards for pigs.

10.1128/mSystems.00176-20.5 TABLE S1 Dietary composition and nutrient levels (as-fed basis). Download Table S1, DOCX file, 0.02 MB. Copyright © 2020 Pi et al. 2020 Pi et al. This content is distributed under the terms of the Creative Commons Attribution 4.0 International license.

### Sample collection.

The timeline of the experiment can be seen in Fig. S4. At day 19 of the experiment, the pigs were fasted for 12 h and then weighed, and blood samples (10 ml) were collected from the jugular vein prior to slaughter. Blood samples were centrifuged at 3,000 × *g* for 15 min at 4°C to separate the serum for chemical analysis. Within 5 min after slaughter, the colon was carefully excised and fastened in length and then weighed both with and without contents. Then, segments of the distal colon tissue were collected and washed three times in ice-cold phosphate-buffered saline. Mucosal samples were then scraped from the underlying tissue using a sterile glass slide, immediately transferred into liquid N, and then stored at −80°C. Representative samples of colonic digesta were collected, and the first part of each sample was diluted with twice the volume of distilled water, mixed, and immediately centrifuged at 2,000 × *g* for 10 min. The supernatants were stored at −80°C for analysis of microbial metabolites. The second part of the digesta samples were placed in a 65°C oven for 48 h and then shattered and stored at −20°C for nitrogen substrate detection. In addition, pH was measured by placing the pH probe within the residual proximal colonic digesta using a portable pH meter.

10.1128/mSystems.00176-20.4 FIG S4 The timeline of the experiment. Download FIG S4, TIF file, 2.8 MB. Copyright © 2020 Pi et al. 2020 Pi et al. This content is distributed under the terms of the Creative Commons Attribution 4.0 International license.

### Cell culture.

Human colorectal adenocarcinoma epithelial cells (Caco-2) were cultivated in Dulbecco modified Eagle medium (DMEM)–F-12 supplemented with 10% (vol/vol) fetal bovine serum (FBS) (Gibco, Grand Island, NY, USA), penicillin (100 U/ml), and streptomycin (100 mg/ml) at 37°C and in a 95% air-5% CO_2_ atmosphere. The effects of bile acids (CA, DCA, and LCA) on gene expression for epithelial barrier function were tested with dosages (25, 100, and 250 μM) based on a previous study (41). Caco-2 cells were seeded onto collagen-coated 9-mm petri dishes at a density of 5.95 × 10^4^ cells per cm^2^ and maintained at 37°C, 5% CO_2_, for 10 days (i.e., for the same incubation time as cells used for paracellular permeability). Culture medium was changed every 48 h. For inhibitor studies, cells were pretreated with inhibitors for 2 h, and the concentrations of inhibitors were as follows according to the previous reports: 250 nM EGFR inhibitor AG-1478 (43), 10 μM Src inhibitor PP-2 (MedChem Express, USA) (23). Cells were plated at a density of 1.0 × 10^4^ cells/well in 96-well plates and treated with inhibitor for 24 h. Dimethyl sulfoxide (DMSO)-methanol at equimolar concentrations to AG-1478 and PP-2 concentrations served as vehicle control.

### Cell viability.

Cell viability was determined using the CCK-8 assay (Nanjing Jiancheng Institute of Bioengineering, Nanjing, China) according to the manufacturer’s protocol. Briefly, Caco-2 cells were plated in 96-well culture plates at a density of 1.0 × 10^4^ cells/well 24 h before treatment. Then, the cells were treated with 0, 25, 100, and 250 μM BAs (CA, DCA, and LCA) in serum-free DMEM for about 2 h. Each cell viability value was normalized to the control and then calculated as previously described (44).

### Transepithelial electrical resistance (TEER) measurements.

To assess paracellular permeability, Caco-2 cells were seeded onto the apical surface of rat-tail collagen (Corning, NY, USA)-coated polyethylene terephthalate cell culture inserts (pore size 0.4 μm, surface area 1.12 cm^2^), which were kept in 12-well companion plates (Becton Dickinson, Falcon labware; Oxford, United Kingdom). Caco-2 cells were seeded at 2.5 × 10^5^ cells in each insert (i.e., 5.95 × 10^4^ cells per cm^2^), and standard growth medium (1.5 ml) was added to each well. Two inserts were cell free with medium only to account for resistance due to the insert membrane. Medium was changed in all inserts every 48 h. Transepithelial electrical resistance (TEER) value was monitored by a Millicell ERS meter (Millipore, Bedford, MA, USA).

### Colonic mucosal bacterial composition analysis.

Total genomic DNA from mucosal scrapings was extracted from 0.3 g of sample using a bead-beating and phenol-chloroform extraction method as described previously (45). The V3-V4 regions of the bacterial 16S rRNA gene were amplified using a universal forward primer (5′-ACTCCTRCGGGAGGCAGCAG-3′) and a reverse primer (5′-GGACTACCVGGGTATCTAAT-3′). Amplicons were purified using an AxyPrep DNA gel extraction kit according to the manufacturer’s instructions (Axygen Biosciences, Union City, CA, USA) and performed on the Illumina MiSeq high-throughput sequencing platform. Total bacterial and functional microbial genes including *baiJ* and *bsh* involved in conversion of primary to secondary BAs were quantified by real-time quantitative PCR (qPCR) using group-specific primers (Table S2) and SYBR green premix (TaKaRa Biotechnology, Dalian, China) in the Step One Plus qPCR system (Life Technologies Inc., CA, USA) as previously described (46).

10.1128/mSystems.00176-20.6 TABLE S2 Primer pairs for host genes. Download Table S2, DOCX file, 0.02 MB. Copyright © 2020 Pi et al. 2020 Pi et al. This content is distributed under the terms of the Creative Commons Attribution 4.0 International license.

10.1128/mSystems.00176-20.7 TABLE S3 Effects of corn starch or casein hydrolysate infusion on growth performance of pigs. Download Table S3, DOCX file, 0.02 MB. Copyright © 2020 Pi et al. 2020 Pi et al. This content is distributed under the terms of the Creative Commons Attribution 4.0 International license.

10.1128/mSystems.00176-20.8 TABLE S4 Effects of corn starch or casein hydrolysate infusion on colonic variables of pigs. Download Table S4, DOCX file, 0.02 MB. Copyright © 2020 Pi et al. 2020 Pi et al. This content is distributed under the terms of the Creative Commons Attribution 4.0 International license.

10.1128/mSystems.00176-20.9 TABLE S5 Relative abundance of dominant bacteria at genus level (% of total sequences). Download Table S5, DOCX file, 0.02 MB. Copyright © 2020 Pi et al. 2020 Pi et al. This content is distributed under the terms of the Creative Commons Attribution 4.0 International license.

10.1128/mSystems.00176-20.10 TABLE S6 Relative abundance of dominant bacteria at OTU level (% of total sequences) in colonic mucosa. Download Table S6, DOCX file, 0.02 MB. Copyright © 2020 Pi et al. 2020 Pi et al. This content is distributed under the terms of the Creative Commons Attribution 4.0 International license.

Raw sequence data generated from MiSeq sequencing were processed in the Mothur version 1.36.1 software (47). Sequences with lengths of <200 bp, having one or more ambiguous bases, or containing a homopolymer length of ≥8 bp were deleted. Then, sequences were normalized to contain an equal number of sequences in all groups before the sequences were presumptively identified and aligned to the high-quality 16S rRNA bacterial sequence database derived from the bacterial Silva database (Silva version 108). These sequences were used for phylotype analysis at the 97% operational taxonomic unit (OTU) level. Representative sequences from each OTU were taxonomically classified with a confidence level of 90% using the Ribosomal Database Project (RDP) classifier. Bacterial diversity including rarefaction analysis, Shannon and inverse Simpson diversity indices, and ACE and Chao richness estimators was assessed using Mothur (47). Principal-coordinate analysis (PCoA) was conducted based on an unweighted UniFrac distance. An unweighted distance-based analysis of molecular variance (AMOVA) was conducted to assess significant differences among samples using the Mothur program (47).

### Colonic bile acids and nutrient substrate measurements.

The concentrations of CA, CDCA, DCA, and LCA were determined in three replicates for each digesta sample using enzyme-linked immunosorbent assay kits (12) (R&D Systems, Shanghai Enzyme-linked Biotechnology CO., Ltd.) according to the manufacturer’s instructions. Samples of colonic contents were analyzed for dry matter (DM), crude protein (CP) (N × 6.25), Ash, and ether extract (EE) according to AOAC (51) procedures. The content of carbohydrate was calculated as 100 − [(100 − DM) (%) + CP (%) + Ash (%) + EE (%)]. The content of nitrogenous compound was calculated as CP (%)/6.25.

### Serum d-lactate and DAO measurements.

The concentrations of d-lactate and diamine oxidase (DAO) in serum were measured by corresponding assay kits (Nanjing Jiancheng Institute of Bioengineering, Nanjing, China) according to the manufacturer’s instructions.

### RT-qPCR.

Total RNA was extracted from colonic mucosa and Caco-2 cells using TRIzol reagent (TaKaRa Bio, Otsu, Japan), and 1 μg RNA was reverse transcribed using a PrimeScript RT reagent kit with cDNA Eraser (TaKaRa Bio) according to the manufacturer’s instructions. Quantitative reverse transcription-PCR (RT-qPCR) of the target genes was performed using the ABI 7300 real-time PCR system (Applied Biosystems, Foster City, CA, USA) with fluorescence detection of SYBR green dye. All the primers were synthesized by Invitrogen Life Technologies (Invitrogen, Shanghai, China) with their sequences shown in Table S2. The reaction system and PCR conditions used in the present study were referring to a previous study (48). The relative quantifications of gene expressions were analyzed by the cycle threshold (*C_T_*) method described previously (48).

### Western blot analysis.

The frozen colonic samples were powdered under liquid nitrogen and lysed in RIPA buffer (150 mM NaCl, 1% Triton X-100, 0.5% sodium deoxycholate, 0.1% SDS, 50 mM Tris-HCl [pH 7.4]) plus a protease inhibitor (Thermo Fisher Scientific, Rockford, IL). After centrifugation at 13,000 × *g* for 10 min at 4°C, the protein concentration in the supernatant fluid was determined using a bicinchoninic acid (BCA) protein assay kit (Beyotime Biotechnology, Beijing, China). Equal amounts of proteins (60 mg) and prestained protein markers were electrophoresed on SDS-polyacrylamide gels. Proteins were transferred to polyvinylidene difluoride (PVDF) membranes (Millipore, Billerica, MA, USA) and blocked in 5% fat-free milk at room temperature in Tris-Tween-buffered saline (TTBS; 20 mM Tris-150 mM NaCl, pH 7.5, and 0.1% Tween 20) for 1 h. Then, samples were incubated with an antibody for ZO-1, glyceraldehyde-3-phosphate dehydrogenase (GAPDH) (1:1,000 dilution; CST, USA), MUC1, OCLD (1:1,000 dilution; Abcam, Cambridge, United Kingdom), Src, EGFR (1:1,000 dilution; Affinity Biosciences, OH, USA), and β-actin (1:1,500 dilution, Abcam, Cambridge, United Kingdom) at 4°C overnight with gentle rocking. The manufacturer has validated all the primary antibodies for use in swine. After being washed three times with TTBS, the membranes were incubated with horseradish peroxidase-conjugated secondary antibodies (goat anti-rabbit IgG; Thermo Fisher Scientific, Rockford, IL) at 1:5,000 dilution for 1 h at room temperature. Finally, the membranes were washed five times with TTBS and then developed (SuperSignal West Dura extended-duration substrate; Pierce, Rockford, IL). Band densities were detected on chemiluminescence (Applygen Technologies Inc., Beijing, China) and quantified using Alpha Imager 2200 (Alpha Innotech Corporation, CA, USA) software.

### Immunohistochemistry analysis.

Colonic tissue samples were removed, fixed in 4% paraformaldehyde, and embedded in paraffin, and 5-μm-thick sections were cut. Briefly, sections were deparaffinized and washed in phosphate-buffered saline (PBS), soaked in 3% H_2_O_2_ for 10 min, washed in PBS, and incubated with goat serum albumin for 20 min. Sections were then incubated with an antibody for ZO-1, OCLD, and MUC1 (1:1,000 dilution; Abcam, Cambridge, United Kingdom) at 37°C for 2.5 h. The embedded tissues were washed with PBS. After the sections were incubated with biotinylated anti-rabbit IgG and then processed by the horseradish peroxidase-conjugated streptavidin, color was developed in the diaminobenzidine (DAB) substrate solution. The sections were then counterstained with hematoxylin, dehydrated, cleared, and permanently mounted. Each sample was used to prepare four slides, and each slide had four sections. The integral optical density (IOD) of each specimen was analyzed by Image Pro Plus 6.0 software.

### Histological measurements.

Paraformaldehyde-fixed colonic tissues were embedded in paraffin, sectioned (6 μm), and stained with hematoxylin and eosin (H&E) or with periodic acid-Schiff stain (PAS). Measurements of crypt length were made using the 40× lens objective. Three slides per pig were prepared, and two images were captured per slide for a total of 36 replicates per measurement per group. Colonic crypt length and number of PAS-positive cells per crypt length were determined by analysis of 30 to 40 well-oriented crypts per pig (*n* = 8/group). Image Pro Plus 6.0 software was used to measure predefined criteria as previously described (49).

### Statistical analysis.

Statistical analysis of all data was performed using SPSS 20.0 (Chicago, IL, USA). For microbiota analysis, prior to investigating the probability of intergroup differences, normality and homogeneity of variances were studied with a Kolmogorov-Smirnov and Levene test, respectively. If the data were normally distributed and variances were equal, a one-way analysis of variance (ANOVA) with (*post hoc*) Bonferroni test was performed. Otherwise, a Kruskal-Wallis test was applied, and *P* values were corrected with the false-discovery rate (FDR) using the Benjamini-Hochberg method (*q* value < 0.05). The FDR *q* value < 0.05 was considered statistically significant. For data on nutrient substrates, microbial metabolites, gene expression, protein expression, and cell numbers, a one-way ANOVA with (*post hoc*) Bonferroni test was also performed, and a *P* value of <0.05 was considered statistically significant. Correlation analysis was assessed by Pearson’s correlation test using GraphPad Prism version 5.00 (GraphPad Software, San Diego, CA, USA). Correlation was considered significant when correlation coefficient was >0.4 and significance was *P* < 0.05.

### Data availability.

The raw reads were deposited into the NCBI Sequence Read Archive (SRA) database (accession number SRP174399).
