# Supplementary figures and images for: Increasing the Hindgut Carbohydrate/Protein Ratio by Cecal Infusion of Corn Starch or Casein Hydrolysate Drives Gut Microbiota-Related Bile Acid Metabolism To Stimulate Colonic Barrier Function
Source: mSystems. 2020 Jun 2;5(3):e00176-20. doi: 10.1128/mSystems.00176-20 (PMC8534727; doi:10.1128/mSystems.00176-20)

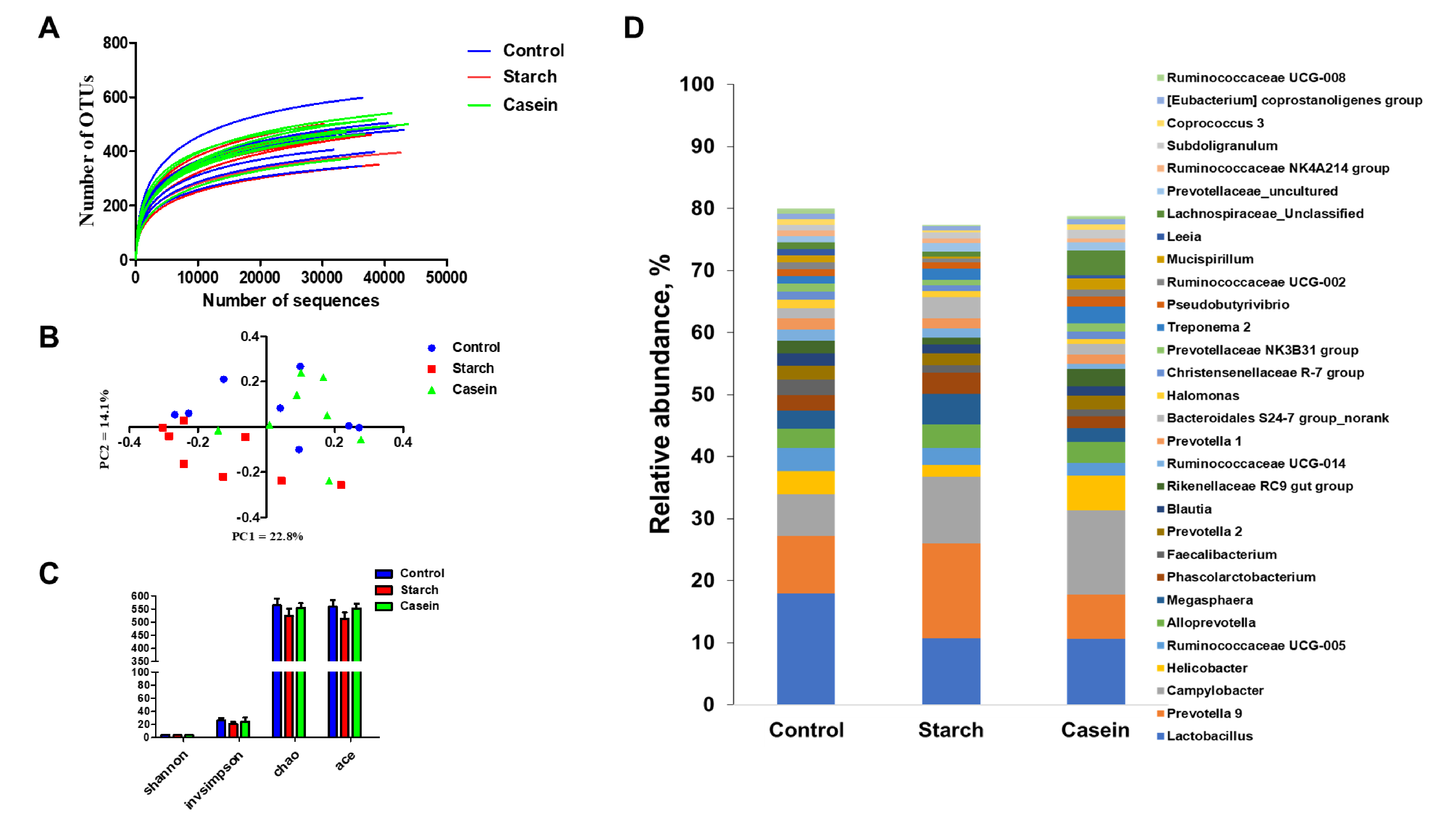

Supplement: FIG S1 [file msystems.00176-20-sf001.tif]

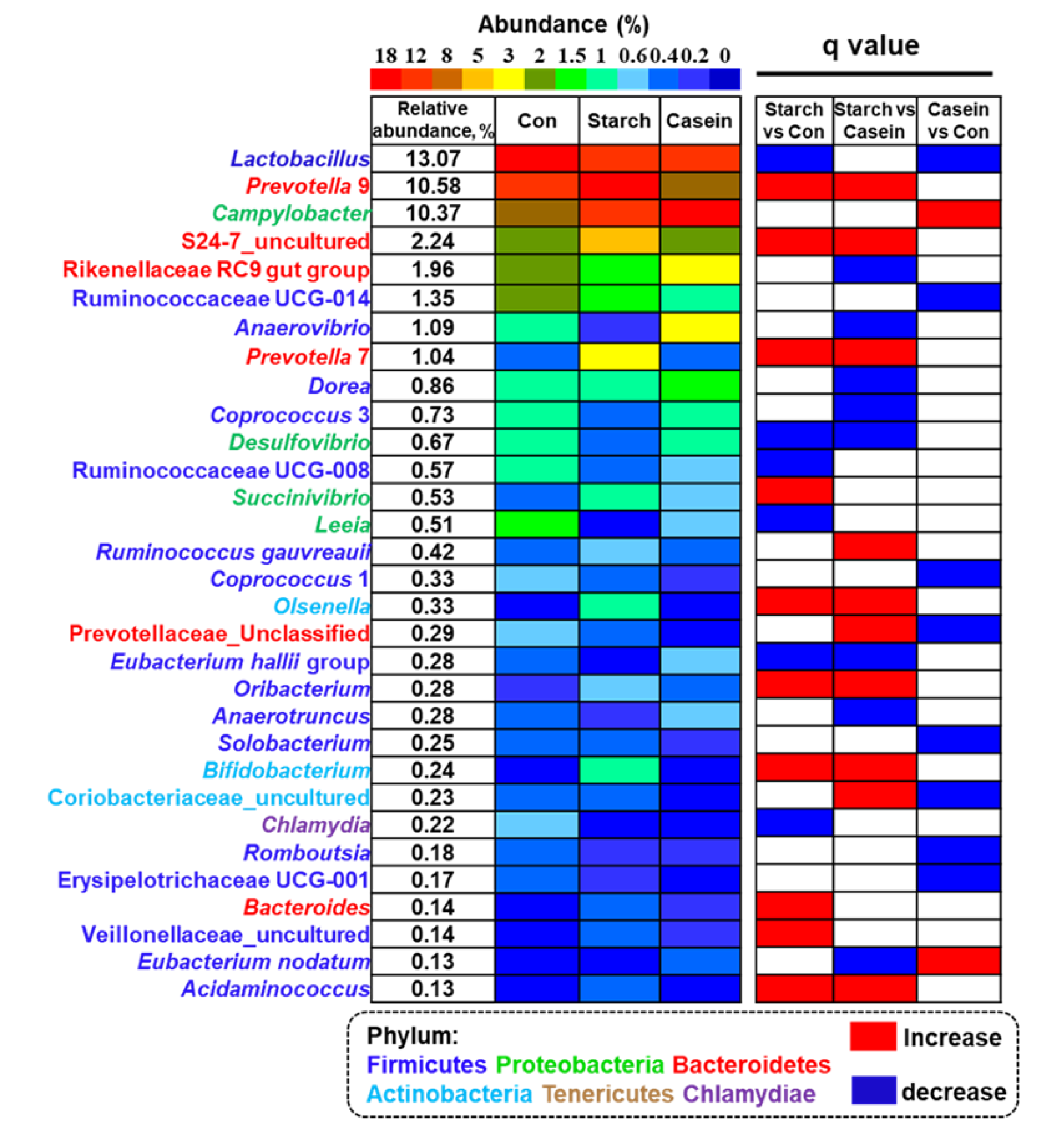

Supplement: FIG S2 [file msystems.00176-20-sf002.tif]

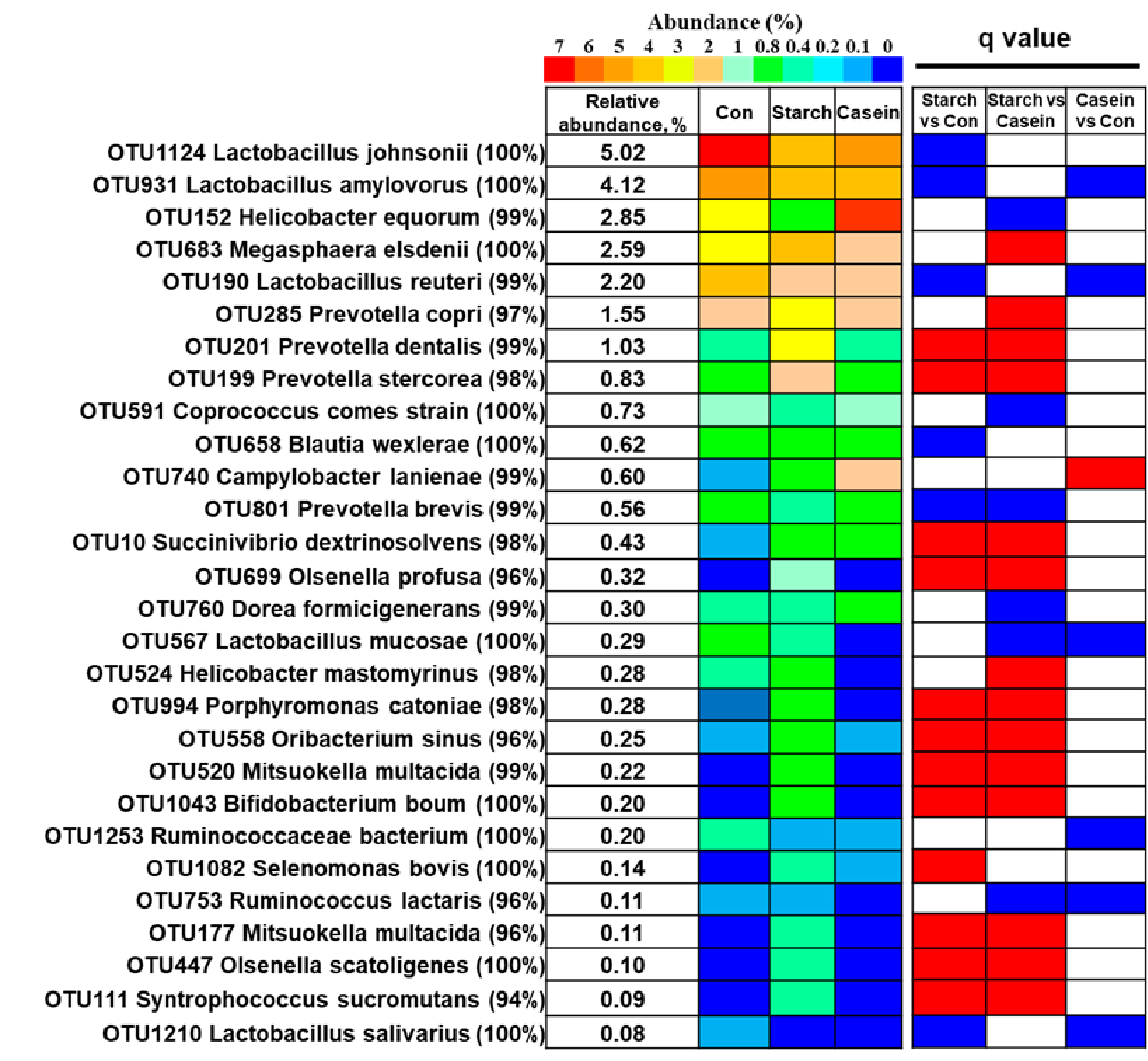

Supplement: FIG S3 [file msystems.00176-20-sf003.tif]

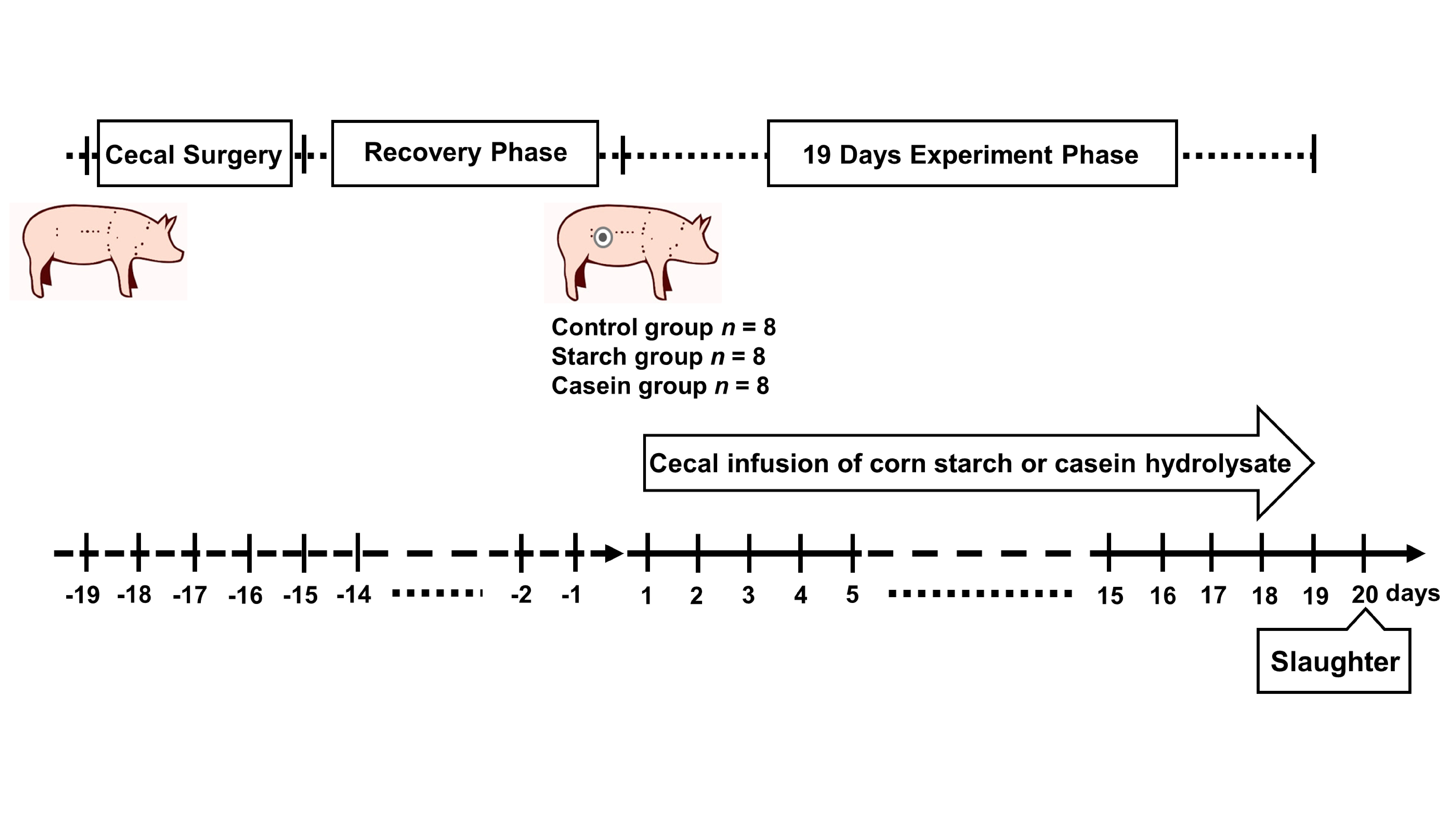

Supplement: FIG S4 [file msystems.00176-20-sf004.tif]
