# Supplementary material for: Increasing the Hindgut Carbohydrate/Protein Ratio by Cecal Infusion of Corn Starch or Casein Hydrolysate Drives Gut Microbiota-Related Bile Acid Metabolism To Stimulate Colonic Barrier Function
Source: mSystems. 2020 Jun 2;5(3):e00176-20. doi: 10.1128/mSystems.00176-20 (PMC8534727; doi:10.1128/mSystems.00176-20)
Supplement: TABLE S1 [file msystems.00176-20-st001.docx]

**Table S1**.

| Items | Composition, % |
| --- | --- |
| Corn (8.0% CP) | 67.7 |
| Soybean meal (45.2% CP) | 20.0 |
| Fish meal (66.2% CP) | 1.00 |
| Limestone | 0.72 |
| Dicalcium phosphate | 1.10 |
| Soy protein concentrate | 2.00 |
| Soybean oil | 2.00 |
| Salt | 0.30 |
| Choline chloride (50%) | 0.10 |
| L-Lysine·HCl (98.5%) | 0.54 |
| DL-Methionine (99%) | 0.23 |
| L-Threonine (98.5%) | 0.21 |
| L-Tryptophan (98%) | 0.05 |
| L-Valine (98.5%) | 0.10 |
| L-Isoleucine (99%) | 0.06 |
| L-Leucine (99%) | 0.13 |
| L-Phenylalanine (99%) | 0.09 |
| L-Histidine·HCl (99%) | 0.07 |
| Fine rice bran | 2.32 |
| Chromic oxide | 0.30 |
| Vitamin and mineral premix^1^ | 1.00 |
| Nutrient levels, % |  |
| NE^2^, MJ/kg | 9.92 |
| CP | 17.42 |
| EE | 4.81 |
| ADF | 4.60 |
| NDF | 14.20 |
| Ash | 4.83 |
| Ca | 0.63 |
| P | 0.53 |
| AA composition^3^, % |  |
| TLys | 1.25 |
| TMet | 0.49 |
| TThr | 0.82 |
| TTrp | 0.24 |
| TVal | 0.89 |
| TIle | 0.71 |
| TLeu | 1.58 |
| TPhe | 0.92 |
| THis | 0.49 |
| TArg | 1.12 |
| TTyr | 0.58 |
| TCys | 0.26 |
| Met+Cys | 0.74 |

^1^ Supplied the following per kg of diet: 8,000 IU, vitamin A; 2400 IU, vitamin D3; 20 mg, vitamin E; 15 mg, pantothenic acid; 5 mg, vitamin B6; 0.3 mg, biotin; 3 mg, folic acid; 0.03 mg, vitamin B12; 40 mg, ascorbic acid; 120 mg, Fe; 25 mg, Cu; 20 mg, Mn; 150 mg, Zn; 0.5 mg, I; 0.30 mg, Se.

^2^ Values for NE were calculated according to Noblet et al., 1994, the contents of EE, CP, ADF, NDF, Ash, Ca, and P were analyzed. NE = net energy.

^3^ T = total
