# Supplementary material for: Increasing the Hindgut Carbohydrate/Protein Ratio by Cecal Infusion of Corn Starch or Casein Hydrolysate Drives Gut Microbiota-Related Bile Acid Metabolism To Stimulate Colonic Barrier Function
Source: mSystems. 2020 Jun 2;5(3):e00176-20. doi: 10.1128/mSystems.00176-20 (PMC8534727; doi:10.1128/mSystems.00176-20)
Supplement: TABLE S2 [file msystems.00176-20-st002.docx]

**Table S2**.

| Target gene | Forward primer (5`-3') | Reverse primer (5'-3') | Annealing temp.(°C) | | Reference |
| --- | --- | --- | --- | --- | --- |
| For host genes of pig | | | | | |
| OCLN | ATGCTTTCTCAGCCAGCGTA | AAG GTTCCATAGCCTCGGTC | | 60 | Zhou *et al*., 2014 |
| ZO-1 | GAGGATGGTCACACCGTGGT | GGAGGATGCTGTTGTCTCGG | | 60 | Zhou *et al*., 2014 |
| ZO-2 | GCAGAGACAACCCCCACTTT | CGTTAACCATGACCACCCGA | | 60 | Alizadeh *et al*., 2015 |
| CLDN1 | TGGCTCCGCGTCTCAGTCC | TGCGAGGGGTGCAGGTCTAA | | 60 | Alizadeh *et al*., 2015 |
| CLDN2 | CTCGTTGGCCTGTATCATCACC | CAGGGGGGAGTAGAAGTCCC | | 60 | Alizadeh *et al*., 2015 |
| CLDN4 | CAACTGCGTGGATGATGAGA | CCAGGGGATTGTAGAAGTCG | | 60 | Pasternak *et al*., 2015 |
| CLDN5 | CCTTCCTGGACCACAACATC | CACCGAGTCGTACACCTTGC | | 60 | Pasternak *et al*., 2015 |
| CLDN7 | TGGTGCTGGGTTTGATG | AAAGACCTGCCACGATG | | 60 | This study |
| MUC1 | ACACCCATGGGCGCTATGT | GCCTGCAGAAACCTGCTCAT | | 60 | Ryan *et al*., 2010 |
| MUC2 | CAACGGCCTCTCCTTCTCTGT | GCCACACTGGCCCTTTGT | | 60 | Ryan *et al*., 2010 |
| MUC4 | GATGCCCTGGCCACAGAA | TGATTCAAGGTAGCATTCATTTGC | | 60 | Ryan *et al*., 2010 |
| MUC12 | GACTAACAAGAATTTCACAAAAGAGCTAA | GCCATCTGAGTCTTGAACTTTTGA | | 60 | Ryan *et al*., 2010 |
| FXR | TATGAACTCAGGCGAATGCCTGCT | ATCCAGATGCTCTGTCTCCGCAAA | | 60 | Radtke *et al*., 2014 |
| TGR5 | CCATGCACCCCTGTTGCT | GGTGCTGTTGGGTGTCATCTT | | 60 | Ipharraguerre *et al*., 2013 |
| EGFR | GCCTTAGCCGTCTTATCCAA | TGGGCACAGATGACTTTGGT | | 60 | Liu *et al*., 2009 |
| *β-actin* | ATGCTTCTAGACGGACTGCG | GTTTCAGGAGGCTGGCATGA | | 60 | Lin *et al*., 2014 |
| *GAPDH* | TTTGCGTCAGTGTCATCG | TGCTCTGCCTTGGGTAAT | | 60 | Fang *et al*., 2014 |
| For host genes of human | | | | | |
| ZO-1 | CAGAGCCTTCTGATCATTCCA | CATCTCTACTCCGGAGACTGC | | 60 | Anderson *et al*., 2010 |
| OCLD | AGGAACCGAGAGCCAGGT | CAATGCCCTTTAGCTTCCAA | | 60 | Anderson *et al*., 2010 |
| EGFR | GTG ACC GTT TGG GAG TTG ATG A | GGC TGA GGG AGG CGT TCT C | | 60 | Chen *et al*., 2006 |
| FXR | GCA GCC TGA AGA GTG GTA CTC TC | CAT TCA GCC AAC ATT CCC ATC TC | | 60 | Casaburi *et al*., 2012 |
| TGR5 | TCG TCT ACT TGG CTC CCA ACT TCT | AGC CCA TAG ACT TCG AGGTAC AGG T | | 60 | Casaburi *et al*., 2012 |
| PI3K | CCACGACCATCATCAGGTGAA | CCTCACGGAGGCATTCTAAAGT | | 60 | Shao *et al*., 2017 |
| RHOA | AGTTTCTTCCGGATGGCAG | CGGTCTGGTCTTCAGCTACC | | 60 | Shao *et al*., 2017 |
| MLCK | CACCGTCCATGAAAAGAAGAGTAG | GAGAGGCCCTGCAGGAAGATGG | | 60 | Shao *et al*., 2017 |
| Src | CGCTGGCCGGTGGAGTGAC | CCAGCTTGCGGATCTTGTAGTGC | | 60 | Lin *et al*., 2015 |
| GAPDH | GGAGCGAGATCCCTCCAAAAT | GGCTGTTGTCATACTTCTCATGG | | 60 | Shao *et al*., 2017 |
| For bacteria |  |  | |  |  |
| Total bacteria | GTGSTGCAYGGYYGTCGTCA | ACGTCRTCCMCNCCTTCCTC | | 60 | Maeda *et al*., 2003 |
| *baiJ* | TCAGGACGTGGAGGCGATCCA | TACRTGATACTGGTAGCTCCA | | 60 | Yoshimoto *et al*., 2013 |
| *bsh* | ATGGGCGGACTAGGATTACC | TGCCACTCTCTGTCTGCATC | | 54 | Duary *et al*., 2012 |

ZO-1, zonula occludens-1; ZO-2, zonula occludens-2; MUC, Mucin; CLDN, Claudin; OCLN, Occludin; GAPDH, glyceraldehyde-3-phosphate dehydrogenase; EGFR, epithelial growth factor receptor; FXR, farnesoid X receptor; TGR5, G-coupled protein receptor; PI3K, phosphoinositide 3-kinase; RHOA, ras homolog gene family, member A; MLCK, myosin light chain kinase; *baiJ*, genes encoding bile salt 7α-dehydroxylase; *bsh*, genes encoding bile salt hydrolase.

Supplementary References：

Lin M, Zhang BL, Yu CN, Li JL, Zhang L, Sun H, Gao F, Zhou GH. 2014. L-Glutamate supplementation improves small intestinal architecture and enhances the expressions of jejunal mucosa amino acid receptors and transporters in weaning piglets. Plos One 9: e111950. https://doi.org/10.1371/journal.pone.0111950.

Fang LD, Jiang X, Su Y, Zhu WY. 2014. Long-term intake of raw potato starch decreases back fat thickness and dressing percentage but has no effect on the longissimus muscle quality of growing-finishing pigs. Livest Sci 170:116-123. https://doi.org/10.1016/j.livsci.2014.10.004.

Zhou XL, Kong XF, Lian GQ, Blachier F, Geng MM, Yin YL. 2014. Dietary supplementation with soybean oligosaccharides increases short-chain fatty acids but decreases protein-derived catabolites in the intestinal luminal content of weaned Huanjiang mini-piglets. Nutr. Res 34:780-788. https://doi.org/10.1016/j.nutres.2014.08.008.

Pasternak JA, Kent-Dennis C, Van Kessel AG, Wilson HL. 2015. Claudin-4 undergoes age-dependent change in cellular localization on pig jejunal villous epithelial cells, independent of bacterial colonization. Mediat Inflamm 2015: 263629. http://dx.doi.org/10.1155/2015/263629.

Alizadeh A, Braber S, Akbari P, Garssen J, Gremmels JF. 2015. Deoxynivalenol impairs weight gain and affects markers of gut health after low-dose, short-term exposure of growing pigs. Toxins 7: 2071-2095. https://doi.org/10.3390/toxins7062071.

Ryan MT, Smith AG, O'Doherty JV, Bahar B, Reilly P, Sweeney T. 2010. Effects of nutrient supplementation with laminarin derived from Laminaria hyperborea and Laminaria digitata on mucin gene expression in the porcine ileum. Livest Sci 133: 236-238. https://doi.org/10.1016/j.livsci.2010.06.074.

Radtke J, Geissler S, Schutkowski A, Brandsch C, Kluge H, Duranti MM, Keller S, Jahreis G, Hirche F, Stangl GL. 2014. Lupin protein isolate versus casein modifies cholesterol excretion and mRNA expression of intestinal sterol transporters in a pig model. Nutr Metab 11: 9. doi:10.1186/1743-7075-11-9.

Ipharraguerre IR, Tedó G, Menoyo D, Cabero NDD, Holst JJ, Nofrarias M, Mereu A, Burrin DG. 2013. Bile acids induce glucagon-like peptide 2 secretion with limited effects on intestinal adaptation in early weaned pigs. J Nutr 143: 1899-1905. doi:10.3945/jn.113.177865.

Liu F, Yin J, Du M, Yan P, Xu J, Zhu X, Yu J. 2009. Heat-stress-induced damage to porcine small intestinal epithelium associated with downregulation of epithelial growth factor signaling. J Anim Sci 87: 1941-1949. doi:10.2527/jas.2008-1624.

Anderson RC, Cookson AL, McNabb WC, Park Z, McCann MJ, Kelly WJ, Roy NC. 2010. *Lactobacillus plantarum* MB452 enhances the function of the intestinal barrier by increasing the expression levels of genes involved in tight junction formation. BMC Microbiol 10: 316. https://doi.org/10.1186/1471-2180-10-316.

Chen A, Xu J, Johnson AC. 2006. Curcumin inhibits human colon cancer cell growth by suppressing gene expression of epidermal growth factor receptor through reducing the activity of the transcription factor Egr-1. Oncogene 25: 278-287. https://doi.org/10.1038/sj.onc.1209019.

Casaburi I, Avena P, Lanzino M, Sisci D, Giordano F, Maris P, Catalano S, Morelli C, Andò S. 2012. Chenodeoxycholic acid through a TGR5-dependent CREB signaling activation enhances Cyclin D1 expression and promotes human endometrial cancer cell proliferation. Cell Cycle 11: 2699-2710. https://doi.org/10.4161/cc.21029.

Shao YX, Wolf PG, Guo SS, Guo YM, Gaskins HR, Zhang BK. 2017. Zinc enhances intestinal epithelial barrier function through the PI3K/AKT/mTOR signaling pathway in Caco-2 cells. J Nutr Biochem 43: 18-26. https://doi.org/10.1016/j.jnutbio.2017.01.013.

Lin TY, Fan CW, Maa MC, Leu TH. 2015. Lipopolysaccharide-promoted proliferation of Caco-2 cells is mediated by c-Src induction and ERK activation. Biomedicine (Taipei) 5: 5. doi: 10.7603/s40681-015-0005-x.

Maeda H, Fujimoto C, Haruki Y, Maeda T, Kokeguchi S, Petelin M, Arai H, Tanimoto I, Nishimura F, Takashiba S. 2003. Quantitative real-time PCR using TaqMan and SYBR Green for *Actinobacillus actinomycetemcomitans*, *Porphyromonas gingivalis*, *Prevotella intermedia*, tetQ gene and total bacteria. Fems Immunol Med Mic 39: 81-86. https://doi.org/10.1016/S0928-8244(03)00224-4.

Yoshimoto S, Loo TM, Atarashi K, Kanda H, Sato S, Oyadomari S, Iwakura Y, Oshima K, Morita H, Hattori M, Honda K, Ishikawa Y, Hara E, Honda K. 2013. Obesity-induced gut microbial metabolite promotes liver cancer through senescence secretome. Nature 499: 97-101. doi:10.1038/nature12347.

Duary RK, Batish VK, Grover S. 2012. Relative gene expression of bile salt hydrolase and surface proteins in two putative indigenous Lactobacillus plantarum strains under *in vitro* gut conditions. Mol Biol Rep 39: 2541-52. doi:10.1007/s11033-011-1006-9.
