# Supplementary material for: Increasing the Hindgut Carbohydrate/Protein Ratio by Cecal Infusion of Corn Starch or Casein Hydrolysate Drives Gut Microbiota-Related Bile Acid Metabolism To Stimulate Colonic Barrier Function
Source: mSystems. 2020 Jun 2;5(3):e00176-20. doi: 10.1128/mSystems.00176-20 (PMC8534727; doi:10.1128/mSystems.00176-20)
Supplement: TABLE S3 [file msystems.00176-20-st003.docx]

**Table S3**.

| Items | Treatments | | | *P*-value |
| --- | --- | --- | --- | --- |
|  | Control | Starch | Casein |  |
| Initial BW (kg) | 18.31±0.74 | 18.43±0.73 | 18.46±0.70 | 0.985 |
| Final BW (kg) | 24.97±1.07^b^ | 28.76±1.17^a^ | 27.65±0.75^ab^ | 0.041 |
| ADG (kg/d) | 0.44±0.04 | 0.54±0.05 | 0.48±0.02 | 0.064 |
| ADFI (kg/d) | 0.85±0.04 | 1.00±0.07 | 0.95±0.05 | 0.071 |
| F: G | 1.97±0.10 | 1.84±0.15 | 2.00±0.11 | 0.497 |

ADFI = average daily feed intake; ADG = average daily weight gain; F: G = feed intake: weight gain. Values shown are means ± SEM, n = 8. Control, control group, pigs cecal infusion with saline; Starch, pigs cecal infusion with corn starch; Casein, pigs cecal infusion with casein hydrolysates. In each row values without a common letter significantly differ, *P* < 0.05.
