# Supplementary material for: Increasing the Hindgut Carbohydrate/Protein Ratio by Cecal Infusion of Corn Starch or Casein Hydrolysate Drives Gut Microbiota-Related Bile Acid Metabolism To Stimulate Colonic Barrier Function
Source: mSystems. 2020 Jun 2;5(3):e00176-20. doi: 10.1128/mSystems.00176-20 (PMC8534727; doi:10.1128/mSystems.00176-20)
Supplement: TABLE S4 [file msystems.00176-20-st004.docx]

**Table S4**.

| Items | Treatments | | | *P*-value |
| --- | --- | --- | --- | --- |
|  | Control | Starch | Casein |  |
| pH value | 6.42±0.15 | 6.63±0.06 | 6.51±0.07 | 0.279 |
| Length (m) | 2.82±0.10 | 2.97±0.15 | 2.94±0.10 | 0.652 |
| Weight (kg) | 0.47±0.04^b^ | 0.67±0.07^a^ | 0.49±0.04^b^ | 0.023 |
| Weight/length | 0.17±0.01^b^ | 0.23±0.01^a^ | 0.17±0.01^b^ | 0.003 |
| Relative weight (percentage of final body weight, %) | 1.76±0.09^b^ | 2.49±0.19^a^ | 1.93±0.10^b^ | 0.003 |
| Digesta weight (kg) | 0.40±0.05 | 0.38±0.05 | 0.32±0.05 | 0.575 |
| Digesta moisture content (%) | 77.37±0.85 | 75.84±0.94 | 75.42±0.83 | 0.317 |

Values shown are means ± SEM, n = 8. Control, control group, pigs cecal infusion with saline; Starch, pigs cecal infusion with corn starch; Casein, pigs cecal infusion with casein hydrolysates. In each row values without a common letter significantly differ, *P* < 0.05.
