# Supplementary material for: Increasing the Hindgut Carbohydrate/Protein Ratio by Cecal Infusion of Corn Starch or Casein Hydrolysate Drives Gut Microbiota-Related Bile Acid Metabolism To Stimulate Colonic Barrier Function
Source: mSystems. 2020 Jun 2;5(3):e00176-20. doi: 10.1128/mSystems.00176-20 (PMC8534727; doi:10.1128/mSystems.00176-20)
Supplement: TABLE S5 [file msystems.00176-20-st005.docx]

**Table S5**.

| Phylum | Family | Genus | Abundance (%) | | | *P*-value | *q*-value |
| --- | --- | --- | --- | --- | --- | --- | --- |
|  |  |  | Control | Starch | Casein |  |  |
| Firmicutes | Lactobacillaceae | *Lactobacillus* | 17.93±2.96^a^ | 10.71±1.47^b^ | 10.59±0.67^b^ | 0.004 | 0.017 |
|  | Lachnospiraceae | *Dorea* | 0.76±0.16^ab^ | 0.61±0.07^b^ | 1.20±0.21^a^ | 0.038 | 0.046 |
|  |  | *[Eubacterium] coprostanoligenes group* | 0.90±0.07^a^ | 0.61±0.10^b^ | 0.80±0.10^ab^ | 0.027 | 0.047 |
|  |  | *Coprococcus 3* | 0.90±0.29^ab^ | 0.36±0.07^b^ | 0.93±0.21^a^ | 0.029 | 0.046 |
|  |  | *[Ruminococcus] gauvreauii group* | 0.39±0.06^ab^ | 0.59±0.17^a^ | 0.24±0.03^b^ | 0.023 | 0.047 |
|  |  | *Coprococcus 1* | 0.54±0.11^a^ | 0.32±0.22^ab^ | 0.20±0.03^b^ | 0.042 | 0.048 |
|  |  | *[Eubacterium] hallii group* | 0.34±0.06^a^ | 0.07±0.02^b^ | 0.45±0.08^a^ | 0.001 | 0.033 |
|  |  | *Oribacterium* | 0.18±0.03^a^ | 0.45±0.12^b^ | 0.22±0.05^a^ | 0.043 | 0.047 |
|  |  | *[Eubacterium] nodatum group* | 0.06±0.02^b^ | 0.06±0.02^b^ | 0.28±0.07^a^ | 0.003 | 0.017 |
|  | Ruminococcaceae | Ruminococcaceae UCG-014 | 1.82±0.37^a^ | 1.44±0.80^ab^ | 0.78±0.10^b^ | 0.018 | 0.042 |
|  |  | Ruminococcaceae UCG-008 | 0.89±0.27^a^ | 0.29±0.08^b^ | 0.52±0.13^ab^ | 0.036 | 0.046 |
|  |  | *Anaerotruncus* | 0.21±0.07^ab^ | 0.15±0.04^b^ | 0.47±0.12^a^ | 0.030 | 0.045 |
|  | Erysipelotrichaceae | *Solobacterium* | 0.37±0.06^a^ | 0.22±0.07^ab^ | 0.17±0.02^b^ | 0.042 | 0.049 |
|  |  | *Erysipelotrichaceae UCG-001* | 0.33±0.25^a^ | 0.13±0.04^ab^ | 0.04±0.00^b^ | 0.017 | 0.043 |
|  | Veillonellaceae | *Anaerovibrio* | 0.88±0.38^ab^ | 0.12±0.02^b^ | 2.26±0.96^a^ | 0.013 | 0.036 |
|  |  | *Veillonellaceae_uncultured* | 0.04±0.02^b^ | 0.27±0.07^a^ | 0.12±0.05^ab^ | 0.007 | 0.023 |
|  | Peptostreptococcaceae | *Romboutsia* | 0.30±0.08^a^ | 0.13±0.04^ab^ | 0.06±0.01^b^ | 0.024 | 0.044 |
|  | Acidaminococcaceae | *Acidaminococcus* | 0.05±0.02^b^ | 0.26±0.10^a^ | 0.07±0.03^b^ | 0.034 | 0.045 |
|  | Unclassified Clostridiales | *Mogibacterium* | 0.01±0.00^b^ | 0.01±0.00^b^ | 0.03±0.01^a^ | 0.002 | 0.013 |
| Bacteroidetes | Bacteroidales S24-7 group | *S24-7_ uncultured* | 1.66±0.42^b^ | 3.37±0.40^a^ | 1.69±0.26^b^ | 0.005 | 0.018 |
|  | Rikenellaceae | Rikenellaceae RC9 gut group | 1.94±0.40^ab^ | 1.13±0.16^b^ | 2.81±0.89^a^ | 0.018 | 0.042 |
|  | Prevotellaceae | *Prevotella 9* | 9.25±2.21^b^ | 15.28±2.42^a^ | 7.20±1.98^b^ | 0.034 | 0.044 |
|  |  | *Prevotella 7* | 0.23±0.05^b^ | 2.58±0.74^a^ | 0.30±0.10^b^ | 0.001 | 0.017 |
|  |  | Prevotellaceae_Unclassified | 0.42±0.21^a^ | 0.38±0.08^a^ | 0.07±0.01^b^ | 0.003 | 0.014 |
|  | Bacteroidaceae | *Bacteroides* | 0.05±0.01^b^ | 0.26±0.09^a^ | 0.12±0.05^ab^ | 0.049 | 0.049 |
| Proteobacteria | Helicobacteraceae | *Campylobacter* | 6.78±1.92^b^ | 10.79±4.12^ab^ | 13.55±3.20^a^ | 0.023 | 0.047 |
|  | Desulfovibrionaceae | *Desulfovibrio* | 0.82±0.24^a^ | 0.31±0.04^b^ | 0.81±0.08^a^ | 0.033 | 0.047 |
|  | Succinivibrionaceae | *Succinivibrio* | 0.28±0.11^b^ | 0.83±0.10^a^ | 0.47±0.20^ab^ | 0.028 | 0.046 |
|  | Neisseriaceae | *Leeia* | 1.04±0.87^a^ | 0.00±0.00^b^ | 0.48±0.44^ab^ | 0.045 | 0.048 |
| Actinobacteria | Coriobacteriaceae | *Olsenella* | 0.01±0.00^b^ | 0.96±0.29^a^ | 0.02±0.00^b^ | 0.001 | 0.011 |
|  |  | Coriobacteriaceae_uncultured | 0.24±0.07^a^ | 0.37±0.15^a^ | 0.08±0.02^b^ | 0.012 | 0.036 |
|  | Bifidobacteriaceae | *Bifidobacterium* | 0.03±0.01^b^ | 0.67±0.23^a^ | 0.04±0.02^b^ | 0.002 | 0.013 |
| Chlamydiae | Chlamydiaceae | *Chlamydia* | 0.58±0.34^a^ | 0.00±0.00^b^ | 0.07±0.06^ab^ | 0.049 | 0.050 |

Only result obtained for the predominant bacterial taxa that were significantly affected by treatments (*q* < 0.05) are presented. Values shown are means ± SEM, n = 8. Control, control group, pigs cecal infusion with saline; Starch, starch group, pigs cecal infusion with corn starch; Casein, casein group, pigs cecal infusion with casein hydrolysates. In each row values without a common letter significantly differ, *q* < 0.05.
