# Supplementary material for: Increasing the Hindgut Carbohydrate/Protein Ratio by Cecal Infusion of Corn Starch or Casein Hydrolysate Drives Gut Microbiota-Related Bile Acid Metabolism To Stimulate Colonic Barrier Function
Source: mSystems. 2020 Jun 2;5(3):e00176-20. doi: 10.1128/mSystems.00176-20 (PMC8534727; doi:10.1128/mSystems.00176-20)
Supplement: TABLE S6 [file msystems.00176-20-st006.docx]

**Table S6**.

| Items | abundance, % | | | *P*-value | *q*-value |
| --- | --- | --- | --- | --- | --- |
|  | Control | Starch | Casein |  |  |
| *OTU111 Syntrophococcus sucromutans (94%)* | 0.02±0.01^b^ | 0.23±0.05^a^ | 0.03±0.02^b^ | 0.001 | 0.009 |
| *OTU10 Succinivibrio dextrinosolvens (98%)* | 0.13±0.03^b^ | 0.77±0.09^a^ | 0.40±0.20^b^ | 0.008 | 0.022 |
| *OTU1082 Selenomonas bovis (100%)* | 0.03±0.02^b^ | 0.27±0.07^a^ | 0.11±0.05^ab^ | 0.008 | 0.020 |
| *OTU1253 Ruminococcaceae bacterium (100%)* | 0.34±0.09^a^ | 0.15±0.06^ab^ | 0.10±0.02^b^ | 0.035 | 0.049 |
| *OTU753 Ruminococcus lactaris (96%)* | 0.14±0.05^a^ | 0.16±0.10^a^ | 0.02±0.01^b^ | 0.017 | 0.039 |
| *OTU199 Prevotella stercorea (98%)* | 0.65±0.20^b^ | 1.14±0.17^a^ | 0.71±0.21^b^ | 0.039 | 0.046 |
| *OTU201 Prevotella dentalis (99%)* | 0.22±0.05^b^ | 2.57±0.74^a^ | 0.30±0.10^b^ | 0.001 | 0.007 |
| *OTU285 Prevotella copri (97%)* | 1.22±0.34^ab^ | 2.35±0.38^a^ | 1.09±0.48^b^ | 0.027 | 0.048 |
| *OTU801 Prevotella brevis (99%)* | 0.71±0.32^a^ | 0.27±0.14^b^ | 0.69±0.13^a^ | 0.033 | 0.048 |
| *OTU994 Porphyromonas catoniae (98%)* | 0.09±0.03^b^ | 0.73±0.20^a^ | 0.02±0.01^b^ | 0.001 | 0.005 |
| *OTU558 Oribacterium sinus (96%)* | 0.16±0.03^b^ | 0.41±0.12^a^ | 0.18±0.05^b^ | 0.052 | 0.056 |
| *OTU447 Olsenella scatoligenes (100%)* | 0.00±0.00^b^ | 0.29±0.15^a^ | 0.00±0.00^b^ | 0.000 | 0.000 |
| *OTU699 Olsenella profusa (96%)* | 0.01±0.00^b^ | 0.96±0.29^a^ | 0.00±0.00^b^ | 0.000 | 0.000 |
| *OTU520 Mitsuokella multacida (99%)* | 0.01±0.00^b^ | 0.62±0.24^a^ | 0.04±0.01^b^ | 0.024 | 0.048 |
| *OTU177 Mitsuokella multacida (96%)* | 0.00±0.00^b^ | 0.28±0.11^a^ | 0.03±0.02^b^ | 0.007 | 0.024 |
| *OTU683 Megasphaera elsdenii (100%)* | 2.24±0.91^ab^ | 3.82±0.78^a^ | 1.72±0.49^b^ | 0.040 | 0.048 |
| *OTU1210 Lactobacillus salivarius (100%)* | 0.19±0.16^a^ | 0.04±0.02^b^ | 0.00±0.00^b^ | 0.001 | 0.005 |
| *OTU190 Lactobacillus reuteri (99%)* | 4.11±1.25^a^ | 1.50±0.87^b^ | 1.47±1.13^b^ | 0.035 | 0.046 |
| *OTU931 Lactobacillus amylovorus (100%)* | 4.97±1.70^a^ | 3.83±0.93^b^ | 3.57±0.99^b^ | 0.023 | 0.049 |
| *OTU567 Lactobacillus mucosae (100%)* | 0.54±0.14^a^ | 0.29±0.09^ab^ | 0.05±0.01^c^ | 0.007 | 0.021 |
| *OTU1124 Lactobacillus johnsonii (100%)* | 7.75±1.41^a^ | 3.54±0.75^b^ | 4.57±1.07^ab^ | 0.035 | 0.044 |
| *OTU524 Helicobacter mastomyrinus (98%)* | 0.38±0.25^ab^ | 0.47±0.26^a^ | 0.00±0.00^b^ | 0.029 | 0.050 |
| *OTU152 Helicobacter equorum (99%)* | 2.70±1.34^ab^ | 0.41±0.09^b^ | 5.44±2.16^a^ | 0.029 | 0.047 |
| *OTU760 Dorea formicigenerans (99%)* | 0.27±0.06^ab^ | 0.21±0.12^b^ | 0.43±0.24^a^ | 0.076 | 0.076 |
| *OTU591 Coprococcus comes strain (100%)* | 0.90±0.29^ab^ | 0.36±0.08^b^ | 0.93±0.21^a^ | 0.029 | 0.044 |
| *OTU740 Campylobacter lanienae (99%)* | 0.07±0.05^b^ | 0.41±0.24^ab^ | 1.22±0.64^a^ | 0.048 | 0.049 |
| *OTU658 Blautia wexlerae (100%)* | 0.79±0.14^a^ | 0.42±0.06^b^ | 0.64±0.11^ab^ | 0.068 | 0.070 |
| *OTU1043 Bifidobacterium boum (100%)* | 0.01±0.01^b^ | 0.57±0.21^a^ | 0.01±0.01^b^ | 0.001 | 0.004 |

Only result obtained for the predominant bacterial taxa that were significantly affected by treatments (*q* < 0.05) are presented. Values shown are means ± SEM, n = 8. Control, control group, pigs cecal infusion with saline; Starch, starch group, pigs cecal infusion with corn starch; Casein, casein group, pigs cecal infusion with casein hydrolysates. In each row values without a common letter significantly differ, *q* < 0.05.
